# A trichromatic MOF composite for multidimensional ratiometric luminescent sensing†
†Electronic supplementary information (ESI) available: Detailed synthesis, encapsulating and sensing procedures. Additional figures (Fig. S1–S32) and tables (Tables S1 and S2). CCDC 1565928. For ESI and crystallographic data in CIF or other electronic format see DOI: 10.1039/c8sc00021b


**DOI:** 10.1039/c8sc00021b

**Published:** 2018-02-21

**Authors:** He Zhao, Jun Ni, Jian-Jun Zhang, Shu-Qin Liu, Ying-Ji Sun, Huajun Zhou, Yan-Qin Li, Chun-Ying Duan

**Affiliations:** a Chemistry College , Dalian University of Technology , Dalian 116024 , China . Email: zhangjj@dlut.edu.cn; b High Density Electronics Center , University of Arkansas , Fayetteville , Arkansas 72701 , USA . Email: hxz001@uark.edu; c State Key Laboratory of Fine Chemicals , Dalian University of Technology , Dalian , 116024 , China . Email: cyduan@dlut.edu.cn; d State Key Laboratory of Structural Chemistry , Fujian Institute of Research on the Structure of Matter , Chinese Academy of Sciences , Fuzhou , 350002 , China

## Abstract

Low-cost, high-performance luminescent probes with wide application potential have been actively pursued. Conventional luminescent probes, which rely on single or dual emissions responsive to analyte molecules, demonstrate limited sensitivity and selectivity because the single emissions can be easily affected by many non-analyte factors, while the dual emissions can only offer single-ratiometric luminescent sensing. Here we report a white-light-emitting trichromatic MOF composite (**W2**) as the first multidimensional ratiometric luminescent probe. It is facilely synthesized by simultaneously incorporating red- and green-emitting iridium and ruthenium complex cations as encapsulated luminescent modules (ELMs) into a porous blue-emitting MOF *via* ion exchange. Specific volatile organic solvents (VOSs) can cause VOS-dependent changes to the MOF-to-ELM energy transfer efficiencies in **W2**, while nitroaromatic (NAC) vapors intriguingly and unprecedentedly quench the three emissions at different rates, both of which enable visible luminescent sensing. Each VOS can be correlated to a unique combination of the two MOF-to-ELM ratios of emission-peak heights, enabling a two-dimensional (2D) code recognition. Furthermore, the time-dependent evolution of the two ratios upon exposure to selective NAC vapors can be mapped out, achieving the first 3D code recognition. Both the synthetic and sensing strategies can be further implemented to develop low-cost and effective luminescent probes.

## Abstract 2

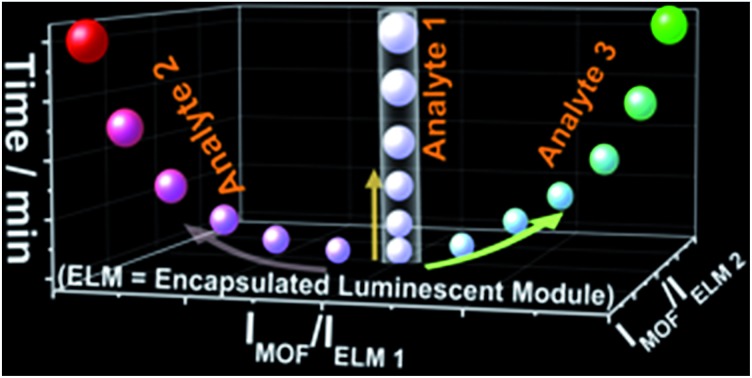
A trichromatic MOF composite utilizes its MOF matrix and two encapsulated cations collectively to achieve unprecedented multi-dimensional ratiometric luminescent sensing with high selectivity and sensitivity.

## Introduction

Luminescent sensing has been proven viable in detecting volatile organic solvents (VOSs), harmful chemicals such as explosive nitroaromatics (NACs) and heavy metal ions.^1^,^2^ Though on-site and selective sensing can sometimes be achieved even accompanied by visible luminescence changes, luminescent sensing still needs significant improvement from the perspectives of selectivity, sensitivity, and cost, before wider application.^3^ To address these issues, recently easily accessible and low-cost metal–organic frameworks (MOFs) have received much attention as new luminescent probes.^4^,^5^ MOFs are built of molecular or supramolecular building blocks (MBBs or SBBs) and multitopic organic linkers. The tremendous options for both the components and their connectivity modes, as well as versatile post-synthetic modifications, can equip MOF-based materials with desired structures, properties, and functionalities.^6^,^7^ For example, their finely regulated porosities can concentrate analyte molecules to amplify the sensing signals and thus increase sensitivity.^8^ They can also provide tailored porosities and environments for analyte molecules to boost selectivity,^9^ and their rich excited-state energy levels can help generate versatile luminescence signals.^10^ All these features make MOF-based materials more advantageous than conventional small molecules in luminescent sensing.

**Scheme 1:**
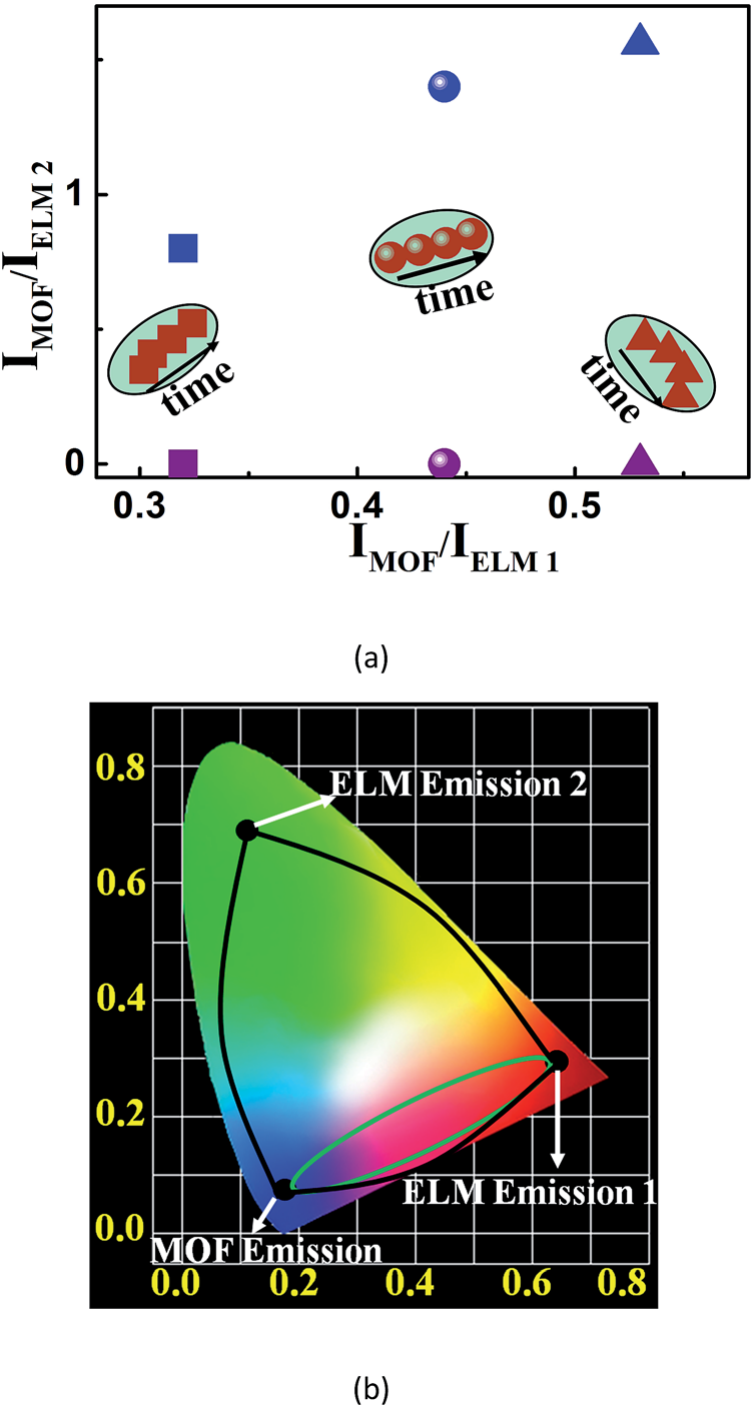
(a) A comparison of 1D (purple), 2D (blue) and 3D (red) code recognition of analytes based on ratiometric luminescent sensing. Different shapes of data points represent different analytes; (b) schematic diagrams illustrating a second difference between 1D and 2D ratiometric luminescent sensing before and during the sensing: the accessible luminescence colours are limited to the intermediates of two emissions as outlined in the green elliptical region in 1D but can reach the whole visible spectrum in 2D sensing.

Up to now, many luminescent MOFs have been used to detect different analytes.^4^,^5^ However, among them, the vast majority output the luminescence intensity evolution of single emissions as their signals when analyte molecules interact with MOFs to turn their emissions on or off. Such monochromatic luminescence signals are not accurate because many non-analyte factors such as unstable instrumental parameters and background luminescence can alter the absolute solid-state luminescence intensities.^11^ Furthermore, such signals are not easily detected by the naked eye. In contrast, sensing based on the luminescence energy changes and visible emission color changes is more sensitive and can be useful in on-site detection. However, such probes are extremely rare because they are generally based on strong host–analyte interactions and thus require elaborate designs for both ligands and frameworks.^12^ Recently an alternative method has involved the incorporation of dyes or Ln^3+^ ions as encapsulated luminescent modules (ELMs) into porous luminescent MOFs to afford dichromatic composites.^13^–^15^ Upon exposure to VOSs, the intensity of the two emissions from both the MOF (*I*_MOF_) and ELM (*I*_ELM_) varies, sometimes accompanied by visible emission color changes; furthermore, a dimensionless and VOS-dependent factor, *i.e.*, *I*_MOF_/*I*_ELM_, is developed to sense VOSs (Scheme 1a). Such one-dimensional (1D) single-ratiometric detection is self-referencing, more reliable and more sensitive than those based on monochromatic MOFs. However, the colors before and during sensing are merely intermediates of the two original colours, resulting in limited color changes and sensing capabilities (Scheme 1b). Therefore, high-quality MOF-based luminescent probes are still being actively sought.

Herein we present a novel white-light-emitting (WLE) MOF composite (**W2**, CIE coordinate: (0.33, 0.34)) using its three tunable red-green-blue emissions for multidimensional ratiometric luminescent sensing. The composite is facilely obtained from the simultaneous incorporation of specific amounts of green- and red-emitting complex cations, namely [Ir(CF_3_-ppy-F_2_)_2_(bpy)]^+^ and [Ru(bpy)_3_]^2+^, into a new blue-emitting MOF (Me_2_NH_2_) [Zn_2_(L) (H_2_O)]·4DMA (**1**) (H_5_L = 2,5-(6-(3-carboxyphenylamino)-1,3,5-triazine-2,4-diyl-diimino)diterephthalic acid); DMA = *N*,*N*-dimethylacetamide) (note: hereafter complex cations [Ir(CF_3_-ppy-F_2_)_2_(bpy)]^+^ and [Ru(bpy)_3_]^2+^ are abbreviated as [Ir]^+^ and [Ru]^2+^). The composition of **W2** can be formulated as **1**⊃(0.28 wt% [Ir]^+^ + 0.45 wt% [Ru]^2+^). The anionic framework in **1** with large cages and channels enables the inclusion of the complex cations while the WLE is achieved by tuning their mole ratios and then emission-intensity ratios. The two MOF-to-ELM ratios of their emission-peak heights, *i.e.*, *I*_MOF_/*I*_ELM 1_ and *I*_MOF_/*I*_ELM 2_, (Scheme 1a) are acquired to achieve two-dimensional (2D) ratiometric luminescent sensing of VOSs and NACs. Such 2D ratiometric luminescent probes are advantageous over the above-mentioned 1D counterparts in three ways. First, the two ratios can be simultaneously changed by an analyte so that the resultant colors can be achieved within the whole visible spectrum and be drastically different from the original white color, enabling the changes to be easily detected by the naked eye (Scheme 1b). Such sensing mechanisms make strong host–analyte interactions unnecessary for effective luminescent sensing and open up great opportunities to develop more capable luminescent probes. Second, a unique 2D code recognition of analytes can be established because each analyte has a characteristic combination of the two ratios. Third, such an approach can be further implemented to record the time-dependent evolution of the two ratios for specific analytes (data points clustered in a region for each analyte are shown in Scheme 1a), adding time as a new dimension and enabling 3D ratiometric luminescent sensing. Obviously, such self-referencing methods double or triple the output information and thus significantly enhance the detection selectivity and sensitivity. In comparison to sensing with a set of three “physically mixed” probes, here the energy transfer between the framework and ELMs can be more effectively tuned by analytes to amplify the changes of the two ratios, leading to much higher sensitivity.

Indeed, WLE **W2** demonstrates the expected sensing results. Upon exposure to certain VOSs and NAC vapours, **W2** undergoes sharp luminescence color changes due to the remarkable change of the two ratios (*i.e.*, *I*_MOF_/*I*_[Ir]_^+^ and *I*_MOF_/*I*_[Ru]_^2+^). Such ratios provide each VOS or NAC with a unique data point, enabling 2D orthogonal identification. Furthermore, this work has allowed the following achievements that have never been reported before to the best of our knowledge. First, NAC vapors intriguingly quench the three emissions in **W2** at different rates; remarkably, *m*-dinitrobenzene (*m*-DNB) vapor is quickly detected (in 1 min) based on dramatic changes of luminescence colors. Second, here the WLE composite is used for ratiometric luminescent sensing of VOSs and NACs. Third, the time-dependent evolution of two ratios upon exposure to NB, *o*-DNB, and *m*-DNB vapors is mapped out, enabling an unprecedented 3D code differentiation of those similar structures.

## Results and discussion

### Structure and characterization of **1**

**Fig. 1:**
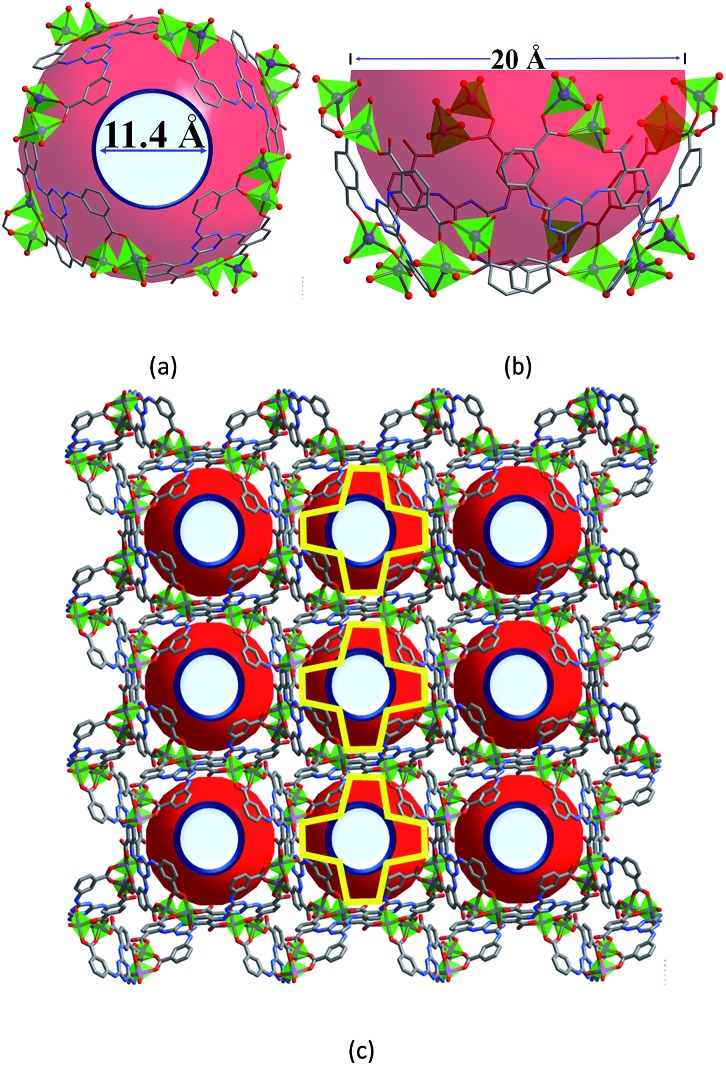
The structure of compound **1**. Top (a) and side (b) views of the bowl-shaped {Zn_16_L_4_} SBB; (c) the framework viewed along the *c* axis; the yellow crosses outline the shapes of the channels.

Colourless crystals of **1** were obtained from a solvothermal reaction of H_5_L and Zn(NO_3_)_2_·6H_2_O in DMA/H_2_O. Single-crystal X-ray diffraction analysis revealed that **1** has a 3D framework based on 3-connected dinuclear quasi-paddlewheel {Zn_2_(COO)_5_} (MBBs) and L^5–^ ligands. The two nodes are connected in a 1 : 1 ratio to build a 3D anionic *bcu-f* network that has a Schläfli symbol of {8;10^2^} according to TOPOS calculation^16^ (Fig. S2†). From another point of view, the framework is built of bowl-shaped {Zn_16_L_4_} SBBs (Fig. 1A and B), each of which is composed of eight {Zn_2_(COO)_5_} MBBs and four L^5–^ ligands, and is 11.4 × 20.0 × 11.73 Å^3^ in dimension. Topologically each SBB is eight-connected, and the structure is a mononodal *bcu* network with its Schläfli symbol being {4^24^;6^4^} (Fig. S3†). The SBBs are packed to afford 1D cross-shaped channels (∼16 Å in length) along the *c* direction. The solvent accessible volume of the guest-free form of **1** is calculated using PLATON^17^ to be about 68.9% of the total volume.

Powder X-ray diffraction (PXRD) analysis confirms the purity and crystallinity of the bulk sample (Fig. S6†). Thermo-gravimetric analysis (TGA) (Fig. S8†) reveals that **1** loses all coordinated water and free DMA solvent molecules before reaching 200 °C (exp. 34.55%; calc. 32.93%). The solid-state luminescence spectra of H_5_L and **1** (*λ*_ex_ = 365 nm) collected at room temperature (Fig. S9†) show the intense emissions of H_5_L and **1** peaked at 470 nm and 416 nm, respectively. The CIE coordinate for **1**’s blue emission is (0.16, 0.07) which is close to that of the saturated blue emitter, (0.14, 0.08).

### Encapsulation of the ELMs and fabrication of the WLE composite

Complex spherical cations [Ir]^+^ and [Ru]^2+^ (structures are shown in Fig. S5†) were chosen as ELMs for two main reasons. First, they are ∼14 and ∼12 Å in diameter, respectively, and both are smaller than the channels in **1**. Second, upon excitation at 365 nm, their solid-state green and red emissions (CIE coordinates: (0.28, 0.52) and (0.55, 0.44), respectively) are much stronger than the blue emission of **1**. Therefore, relatively small amounts (0.28 wt% and 0.45 wt% for [Ir]^+^ and [Ru]^2+^, respectively, in **W2**) of such expensive cations can complement **1** to achieve WLE and leave enough vacant space for further sensing, while saving costs. A similar encapsulation strategy has been recently used to prepare MOF-based composites with luminescent dyes as ELMs for WLE applications.^18^ Compared to organic dyes, noble metal complex cations are better ELMs for ratiometric luminescent sensing due to their higher quantum yields of luminescence, longer lifetime, better photostability and higher sensitivity to external stimuli.^19^

We first found that each complex cation could be individually exchanged into **1** to afford **1**⊃[Ir]^+^ or **1**⊃[Ru]^2+^ when **1** was soaked in a methanol solution of [Ir(CF_3_-ppy-F_2_)_2_(bpy)] (PF_6_) or [Ru(bpy)_3_](PF_6_) for 24 h. The amount of [Ir]^+^ or [Ru]^2+^ included was controlled by varying the solution concentrations, and was accurately determined using luminescence measurements of the filtrates (Fig. S12 and S13†). No cations leaked out from the framework when the cation-exchanged phases were soaked in methanol for an additional 24 h (Fig. S14†), indicating the stabilization of these ELMs, which is possibly due to electrostatic interactions. The PXRD analysis reveals that the resultant phases maintain the original framework (Fig. S7†) while the laser confocal scanning analysis of a single crystal to different depths reveals that these ELMs are uniformly dispersed throughout **1** (Fig. S15 and S16†). Compared to those in their pure phases, the emissions of **1** and of the ELMs in the composites show obvious red and blue shifts, respectively (Fig. S17 and S18†), due to the ‘rigidochromic effect’.^18^ As the concentration of these ELMs increases, the blue emissions from the framework gradually decrease, and the emissions from the ELMs increase along with the blue shifts, both indicating excitation energy transfers from the framework to the ELMs. The efficient resonance energy transfers are also supported by the spectral overlap between the absorption of [Ir(CF_3_-ppy-F_2_)_2_(bpy)]PF_6_/[Ru(bpy)_3_](PF_6_)_2_ and the emission from **1** (Fig. S19†).

**Fig. 2:**
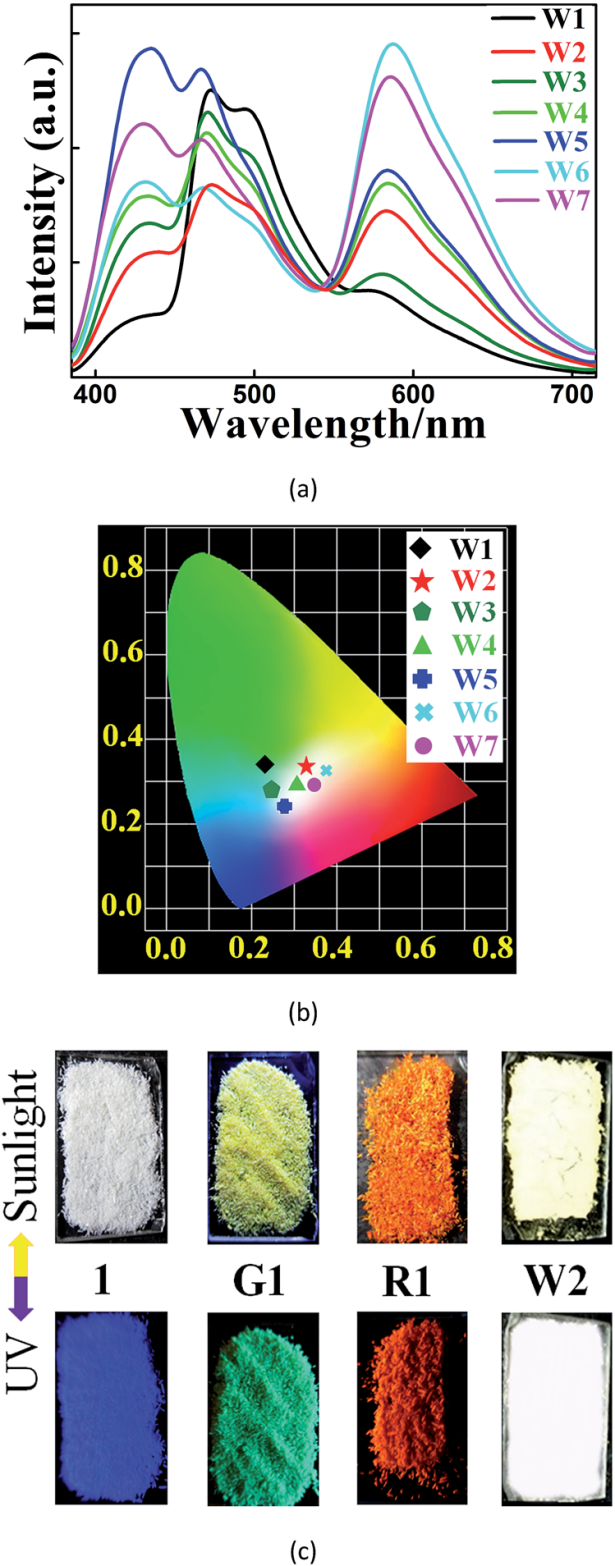
Luminescent spectra (a) and CIE chromaticity coordinates (b) of **1**⊃([Ir]^+^ + [Ru]^2+^) with different amounts of [Ir]^+^ and [Ru]^2+^ (*λ*_ex_ = 365 nm); (**W1**: **1**⊃(0.88 wt% [Ir]^+^ + 0.55 wt% [Ru]^2+^); **W2**: **1**⊃(0.28 wt% [Ir]^+^ + 0.45 wt% [Ru]^2+^); **W3**: **1**⊃(0.18 wt% [Ir]^+^ + 0.28 wt% [Ru]^2+^); **W4**: **1**⊃(0.18 wt% [Ir]^+^ + 0.46 wt% [Ru]^2+^); **W5**: **1**⊃(0.13 wt% [Ir]^+^ + 0.35 wt% [Ru]^2+^); **W6**: **1**⊃(0.065 wt% [Ir]^+^ + 0.46 wt% [Ru]^2+^); **W7**: **1**⊃(0.065 wt% [Ir]^+^ + 0.42 wt% [Ru]^2+^)). (c) Photographic images of **1** and its composites deposited on quartz slides under sunlight and under UV illumination at 365 nm; (**R1**: **1**⊃5.70 wt% [Ru]^2+^; **G1**: **1**⊃5.90 wt% [Ir]^+^).

We further found that both cations could be simultaneously included into **1** when **1** was soaked in a solution containing both cations (Fig. 2). Different WLE composites with different amounts of ELMs can be obtained by tuning their concentrations in the solution. Among them, **W2**, namely **1**⊃(0.28 wt% [Ir]^+^ + 0.45 wt% [Ru]^2+^), displays broadband emission covering the whole visible spectral region with three main emissions peaking at 438, 472 and 580 nm. Its corresponding CIE coordinate is (0.33, 0.34) which is very close to that of the pure WLE, (0.33, 0.33). Its calculated CCT value of 5604 K indicates that it is a positive white-light source. A comparison (Fig. 2C) of the colors of **1** and its composites under ambient conditions and upon UV light irradiation (at 365 nm) further confirms the successful preparation of the composites.

### Sensing of VOSs by the WLE composite **W2**

**Fig. 3:**
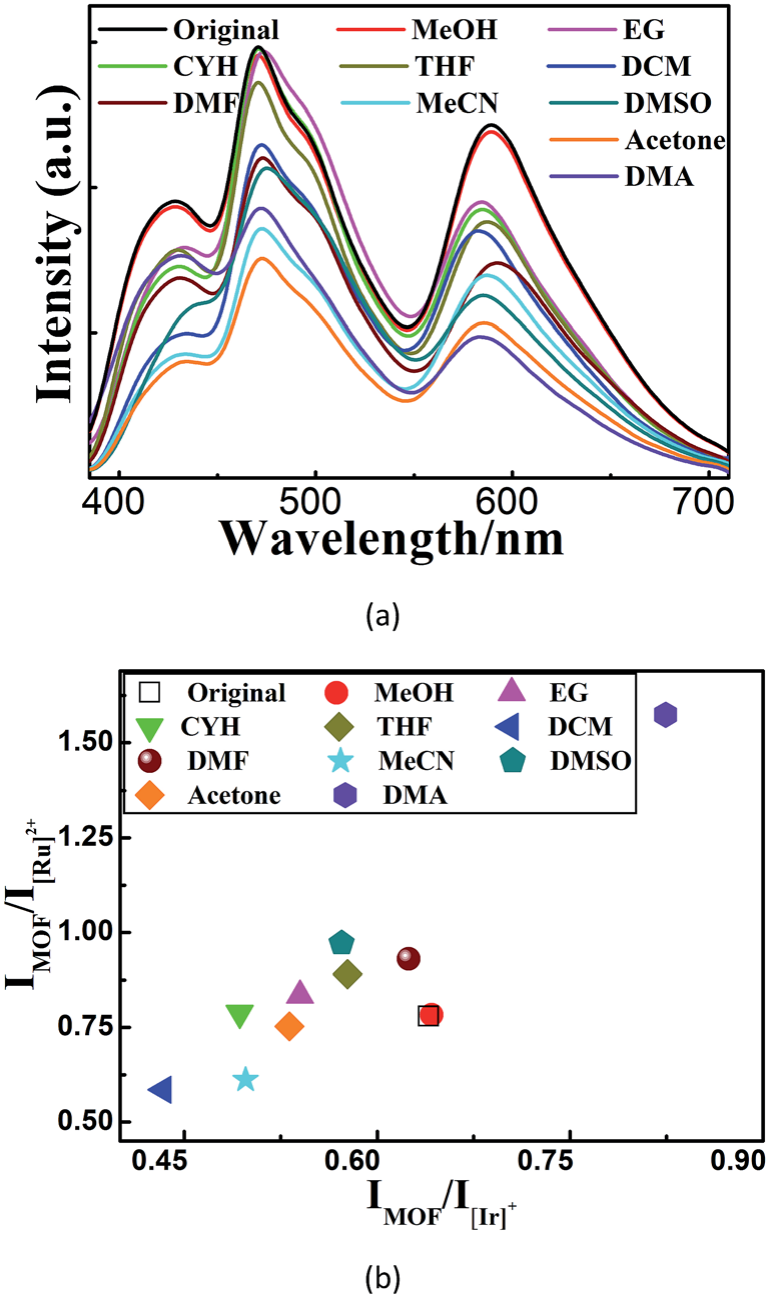
(a–b) The emission spectra (a) and the corresponding 2D decoded map (b) of ten solvent molecules based on two emission intensity ratios: *I*_MOF_/*I*_[Ir]_^+^ and *I*_MOF_/*I*_[Ru]_^2+^.

We then investigated the sensing behavior of **W2** towards various VOSs. From the emission spectra of its suspensions in different VOSs (Fig. 3A), a unique combination of both *I*_MOF_/*I*_[Ir]_^+^ and *I*_MOF_/*I*_[Ru]_^2+^ are derived for each VOS. Such VOS-dependent ratiometric luminescence is caused by the changes of the MOF-to-ELM energy transfer efficiencies and/or the energy allocation between [Ir]^+^ and [Ru]^2+^ when different VOS molecules are included within the MOF channels/cages.^13^ Thus **W2** is a self-calibrating probe using the two ratios instead of using the single absolute emission-peak heights to identify VOSs. This sensing method is cheaper and more accurate and facile than the recently reported dual-readout sensing method that measures both single emission-peak height ratios and luminescence quantum yields using expensive and sophisticated equipment.^20^ It is also different from a few reported dual-ratiometric luminescent sensing methods in which the duality refers to the capabilities of single ratiometric luminescent sensing to detect both pH and temperature.^21^

**Fig. 4:**
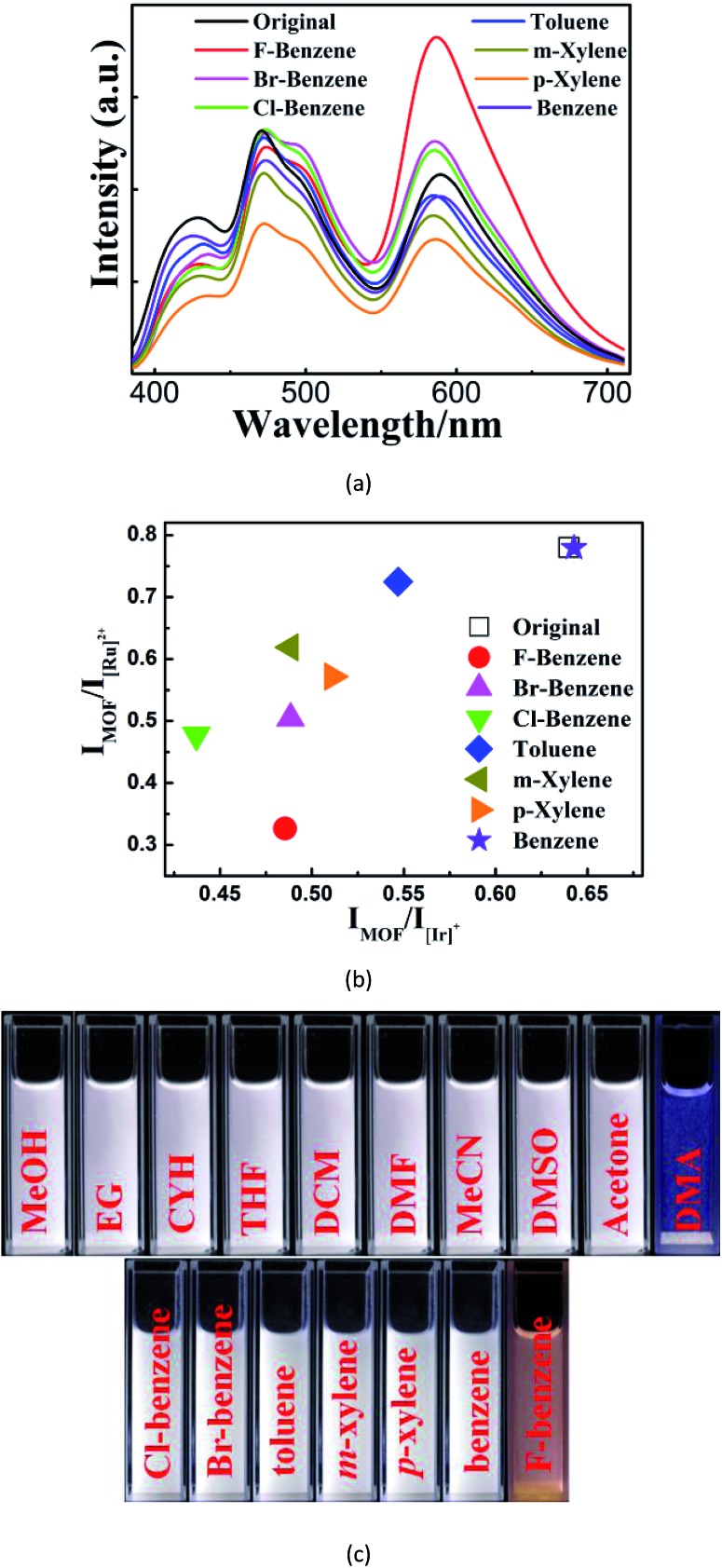
(a–b) The emission spectra (a) and the corresponding 2D decoded map (b) of seven structurally highly similar aromatic molecules based on two emission-intensity ratios: *I*_MOF_/*I*_[Ir]_^+^ and *I*_MOF_/*I*_[Ru]_^2+^; (c) photographic images of the emulsions of **W2** in different solvents under UV light irradiation at 365 nm.

With the advantages mentioned above, **W2** can unambiguously differentiate aromatic molecules with very similar structural motifs, such as *m*- and *p*-xylene, benzene, toluene, bromobenzene (Br-benzene), fluorobenzene (F-benzene), and chlorobenzene (Cl-benzene) (Fig. 4A and B). Remarkably, under illumination at 365 nm, **W2** changed to blue or orange upon exposure to DMA or F-benzene, respectively, while being white or near-white upon exposure to other VOSs. Therefore, **W2** is a selective and sensitive luminescent probe enabling the detection of DMA and F-benzene to be visible to the naked eye (Fig. 4C and S20†), possibly mainly due to the fact that DMA and F-benzene induce larger changes of the two ratios than other VOSs. It is worthy to note that so obvious color changes such as ours were observed in other luminescent sensing MOFs with strong host–analyte interactions^12^ that are not required here.

### Sensing of NACs by the WLE composite **W2**

**Fig. 5:**
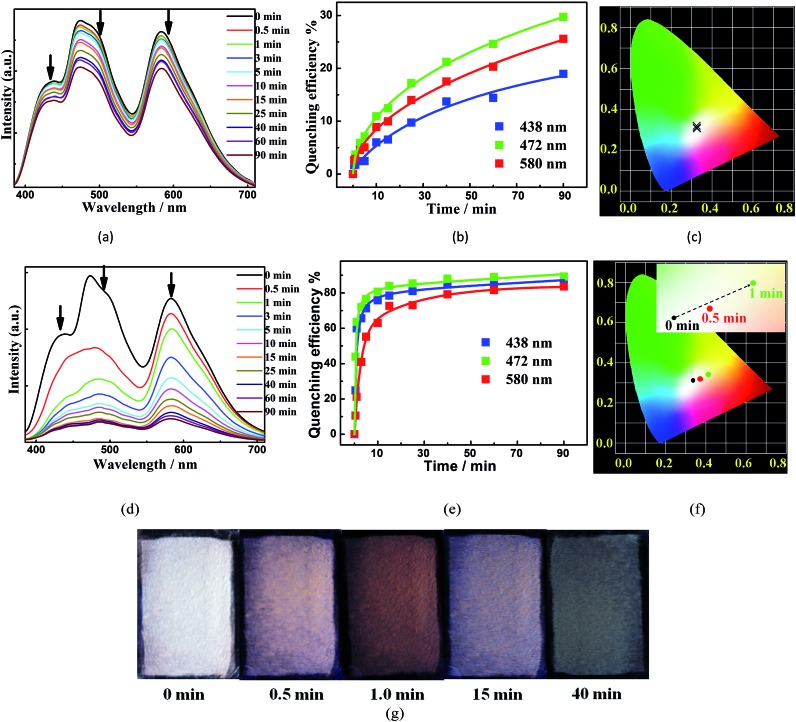
(a–c) Time-dependent evolution of luminescence spectra (a), quenching efficiencies (b), and CIE coordinates (c) of **W2** upon exposure to *m*-DNB vapour at 25 °C. (d–f) Time-dependent evolution of luminescence spectra (d), quenching efficiencies (e), CIE coordinates (f), and photographic images under UV illumination at 365 nm (g) of **W2** upon exposure to *m*-DNB vapour at 60 °C.

We further explored the ability of **W2** to probe NAC vapors by recording the luminescence spectra of its thin films before and immediately after being exposed to the equilibrated vapors of different NACs at 25 °C for 15 min. The fifteen NACs under examination are nitrobenzene (NB), *m*-DNB, *o*-DNB, *p*-DNB, 1-nitrobromobenzene (1-NBB), 3-NBB, 4-NBB, 1-nitrotoluene (1-NT), 3-NT, 4-NT, 1-nitronaphthalene (1-NP), 2-nitrophenol (2-NPHEN), 4-nitrophenol (4-NPHEN), dinoseb, and 2,4,6-trinitro-phenol (TNP). Among them, only the vapors of NB and *m*-DNB (Fig. 5A–C) can obviously quench the three main emissions (438 nm, 472 nm, and 580 nm) and the corresponding quenching efficiencies, calculated from the equation [(*I*_0_ – *I*)/*I*_0_] × 100% (where *I*_0_ and *I* are the initial luminescence intensity and the 15 min luminescence intensity upon exposure, respectively), are 15.4%, 26.9%, and 20.1% for NB, and 6.8%, 12.4%, and 9.8% for *m*-DNB (Fig. S21–S26†). The quenching could be due to the MOF-to-NAC electron transfers (Fig. S31†) and the higher vapor pressures of the two NACs.^22^ Detailed analyses of the CIE coordinates of each spectrum (Fig. 5C) during the quenching process indicate that the CIE coordinates keep changing during the process, possibly mainly due to their different quenching rates upon exposure to the same VOS vapour.

We further increased the sensing temperature to 40 °C and 60 °C to amplify the quenching-rate difference and the corresponding color changes during sensing. Compared to those obtained at 25 °C when **W2** was exposed to *m*-DNB vapors for 15 min, the quenching efficiencies of the three emissions at 60 °C were increased ∼10.4, 5.9 and 6.1 times to reach 77.5%, 85.7%, and 69.3%, respectively (Fig. 5D–F, 15 min of exposure). Furthermore, during the first minute at 60 °C, the *m*-DNB vapor induced the largest quenching-rate difference, and *I*_[Ir]_^+^ was quenched much faster than *I*_[MOF]_ and *I*_[Ru]_^2+^ (Fig. 5D); therefore, **W2** underwent a visible colour change under a standard UV lamp (365 nm) (Fig. 5G) to a distinctive dark orange colour (CIE coordinate: 0.39, 0.35). After that, its color gradually returned but it did not reach the starting white color again. In contrast, NB and *o*-DNB induced a quenching effect at 40 °C and 60 °C to some extent, but the changes of the CIE coordinates were not significant enough to bring about visible luminescence color changes (Fig. S29†). The rapid and visible responses make **W2** an unprecedentedly practical and selective probe for *m*-DNB, representing a significant step forward in reaching instantaneous, highly selective and sensitive detection of NAC vapors. In comparison, until now only a handful of MOFs have been used to sense non-NB NAC vapors, and during the sensing processes single emissions are quenched and turn dark while the changes generally are difficult to detect with the naked eye.^4^,^5^ Moreover, many more MOF probes have been used to sense NACs in solutions with limited success in affording luminescence color changes that are visible to the naked eye.^14^,^23^ Unfortunately, after being quenched during the sensing experiments, the three emissions in **W2** could only be partially regenerated after being washed with MeOH and dried, and thus the original ratiometric sensing behavior cannot be recovered.

**Fig. 6:**
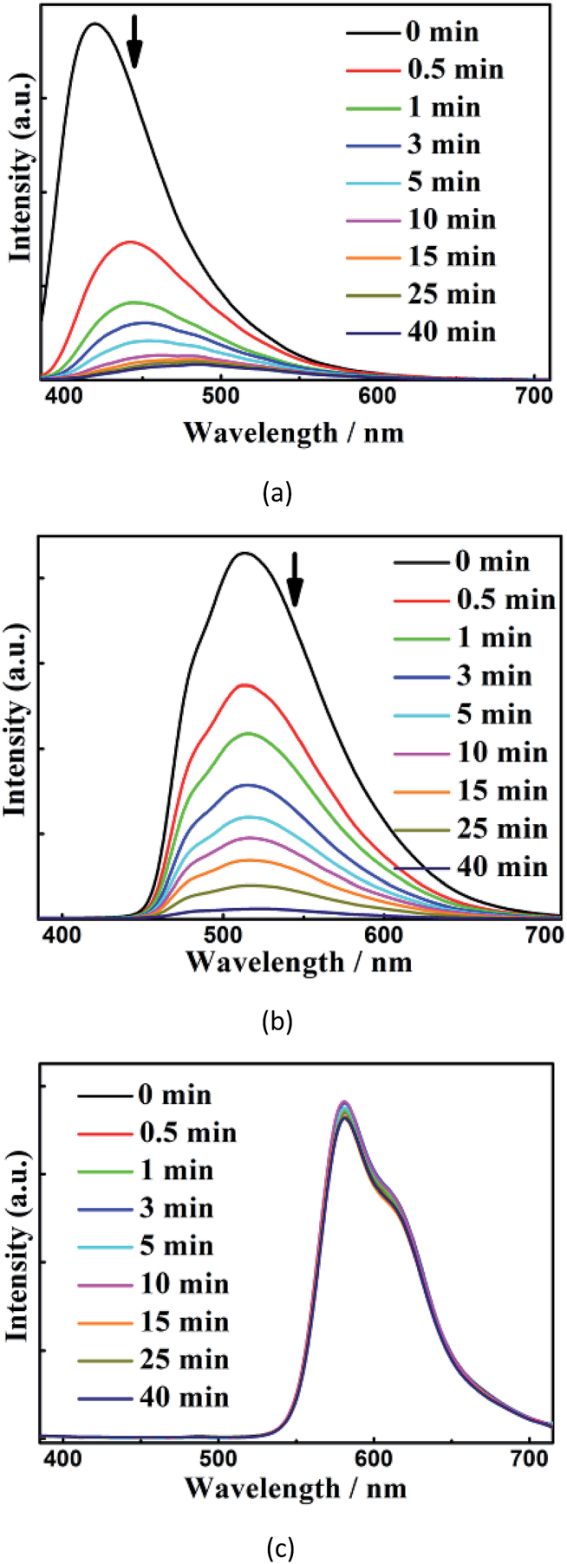
Time-dependent evolution of the luminescence spectra of **1** (a), [Ir(CF_3_-ppy-F_2_)_2_(bpy)]PF_6_ (b), and [Ru(bpy)_3_](PF_6_)_2_ (c) upon exposure to *m*-DNB vapour at 60 °C.

To understand the mechanisms of their different quenching rates,we investigated the individual quenching behaviors of **1**, [Ru(bpy)_3_](PF_6_)_2_ and [Ir(CF_3_-ppy-F_2_)_2_(bpy)]PF_6_ by *m*-DNB at 60 °C (Fig. 6 and S27†). Indeed, *m*-DNB vapor can quench the emissions of **1** and [Ir(CF_3_-ppy-F_2_)_2_(bpy)]PF_6_ but not the emission of [Ru(bpy)_3_](PF_6_)_2_. The significantly changed MOF-[Ru]^2+^ interactions before and upon exposure to *m*-DNB vapor can explain why *m*-DNB vapor can quench the emission from the [Ru]^2+^ ELM in the composite. Before exposure to *m*-DNB vapor, the framework can amplify the ELM emissions in two ways. First, the spectral overlap between the emission from the framework and the absorption of [Ir(CF_3_-ppy-F_2_)_2_(bpy)]PF_6_/[Ru(bpy)_3_](PF_6_)_2_ (Fig. S19†) can enhance the ELM emissions. Second, the framework provides an excellent environment that can restrain the mobile movement of the ELMs and thus significantly suppress the aggregation-induced quenching.^18^ In contrast, in the physical mixture of the three compounds in the same weight ratio, the emissions from [Ru(bpy)_3_](PF_6_)_2_ and [Ir(CF_3_-ppy-F_2_)_2_(bpy)]PF_6_ slightly alter the emission from the framework in the range of 450–700 nm (Fig. S32†). Upon exposure to *m*-DNB vapor, the photo-induced electron transfers from the conduction band (CB) of the electron-rich framework to the lower LUMO orbitals of the electron-deficient *m*-DNB rather than to the ground state with relaxation, which leads to the quenching of the emission from the framework.^22^ Such quenching leads to a significantly reduced MOF-to-[Ru]^2+^ energy transfer and the subsequent quenching of the emission from [Ru]^2+^. This quenching route also applies in the case of [Ir]^+^ that is further directly quenched by *m*-DNB. As a result, the three components have different quenching rates. Investigations toward further understanding of the underlying mechanisms are ongoing.

**Fig. 7:**
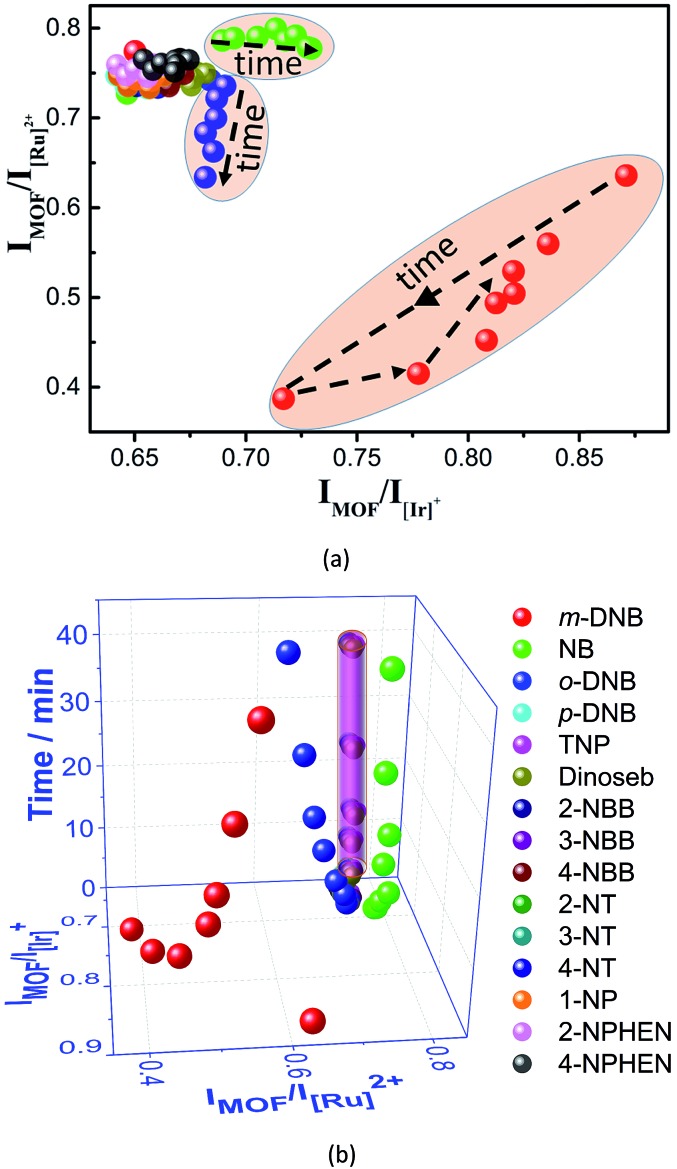
(a) The time-dependent 2D ratiometric sensing of fifteen NAC vapours by **W2** at 60 °C, and (b) the derived 3D ratiometric sensing of the NAC vapours when time is added as the third dimension (note: the overlapped data points from the twelve NAC vapors without quenching effects are combined to form a column).

Interestingly, the luminescent sensing of NAC vapors at 60 °C can be further improved by recording the time-dependent evolution of the two ratios (Fig. 7). For the twelve NACs without quenching inducing behaviors, all their data points (*I*_MOF_/*I*_[Ir]_^+^: 0.64–0.68; *I*_MOF_/*I*_[Ru]_^2+^: 0.73–0.77) during the sensing processes are very close to the original value of (0.650, 0.777) and are densely located in a small region, possibly due to random and/or systematic errors. In contrast, although the data points of NB, *m*-DNB, and *o*-DNB before and during the initial sensing are located in the same region, they move into totally different regions in the subsequent sensing (Fig. 7A). When time is added as the third dimension, a novel 3D code recognition can be mapped out to further distinguish them (Fig. 7B). Here, **W2** identifies a series of analytes with similar structures and luminescence changes that generally cannot be differentiated by probes based on single or dual emissions, providing a new direction for luminescent sensing.

## Conclusions

In summary, we have successfully developed a novel WLE MOF-based composite as the first multidimensional ratiometric luminescent probe. It is facilely obtained by incorporating two luminescent complex cations as ELMs into an anionic, porous and luminescent MOF. Upon exposure to selective VOSs or NAC vapors, the two MOF-to-ELM ratios of emission-peak heights undergo analyte-dependent changes, leading to drastic luminescence color changes and making **W2** a highly sensitive and selective luminescent probe. The novel sensing upgrades current state-of-the-art 1D ratiometric luminescent sensing into 2D and 3D versions, highlighting a new direction for achieving more facile and efficient luminescent sensing. Furthermore, the post-synthetic method described here can be employed to develop more multi-chromatic (*e.g.*, tetrachromatic) probes using numerous available and suitable porous materials (including MOF hosts) and ELM species to achieve even higher dimensional code recognition. In short, this work opens up fresh opportunities to develop new composites for luminescent sensing and related applications.

## Conflicts of interest

There are no conflicts to declare.

## Supplementary Material

Supplementary information

Crystal structure data
