# Supplementary material for: A trichromatic MOF composite for multidimensional ratiometric luminescent sensing
Source: Chem Sci. 2018 Feb 21;9(11):2918–26. doi: 10.1039/c8sc00021b (PMC5914538; doi:10.1039/c8sc00021b)
Supplement: Supplementary file 1 [file SC-009-C8SC00021B-s001.pdf]

Electronic Supplementary Material (ESI) for Chemical Science.
This journal is © The Royal Society of Chemistry 2018

# Supporting Information

## A Trichromatic MOF Composite for Multidimensional Ratiometric Luminescent Sensing

He Zhao, Jun Ni, Jian-Jun Zhang,* Shu-Qin Liu, Ying-Ji Sun, Huajun Zhou,* Yan-Qin Li and Chun-Ying Duan*

# Experimental Procedures

## Materials and physical measurements

All commercially available reagents and solvents were used as received without further purification. Infrared spectra (IR) were recorded on Nicolet-20DXB spectrometer as KBr pellets in the range of 4000−400 $cm^{-1}$. ESI mass spectra were recorded using a LCQ-Tof MS mass spectrometer (Samples were deprotonated using NaAc). NMR spectra were recorded at ambient temperature on a Bruker Avance II 400M spectrometer. Thermogravimetric analyses (TGAs) were performed under nitrogen atmosphere with a heating rate of 10 °C/min using a TA-Q50 thermogravimetric analyzer. Elemental analyses of C, H, and N were determined on a Vario EL Ⅲ Elemental Analyzer. Powder X-ray diffraction patterns (PXRD) data were collected on a D/MAX-2400 X-ray Diffractometer. Luminescent spectra were acquired at ambient temperature by using a PerkinElmer LS55 fluorescence spectrophotometer. $[Ir(CF_3\text{-ppy-}F_2)_2(bpy)]PF_6$[1] and $[Ru(bpy)_3](PF_6)_2$[2] were synthesized according to reported methods. The correlated colour temperature (CCT) values were calculated using the reported equation.[3]

## Synthesis of 2,5-(6-(3-carboxyphenylamino)-1,3,5-triazine-2,4-diyldiimino) diterephthalic acid ($H_5L$)

A solution of cyanuric chloride (4.6 g, 25 mmol) in 25.0 mL of acetone was added drop-wise to an aqueous solution (100 mL) of 2-aminoterephthalic acid (9.0 g, 50 mmol), NaOH (4.0 g, 100 mmol) and $NaHCO_3$ (4.2 g, 50 mmol). The mixture was then stirred at 45 °C for 3.5 h. Excess EtOH was added to form a colorless precipitate which was collected by filtration. The solid was dissolved in 50 mL $H_2O$ then combined with an aqueous solution of 3-aminobenzoic acid (2.74 g, 20 mmol), NaOH (0.8 g, 20 mmol) and $NaHCO_3$ (1.68 g, 20 mmol). The reaction mixture was refluxed at 100 °C for 15 h. After cooling to room temperature, the solution was acidified with 6 M HCl solution until pH = 2 to obtain a colorless precipitate

which was collected by filtration, washed with $H_2O$ ad EtOH and dried in a vacuum desiccator to give 11.6 g (81%) of $H_5L$. mp > 300 ºC. Negative ESI-MS ( m/ z): 573.15 ($H_4L^-$). $^1H$ NMR (DMSO-d6): d 7.38 (t, 1H), 7.61 (d, 3H), 8.06 (d, 3H), 8.13 (s, 1H), 8.17 (s, 1H), 9.11 (s, 1H), 9.94 (s, 1H), 10.87(s, 2H), 13.03(s, 5H). Elemental analysis (%) calcd. for $C_{26}H_{18}N_6O_{10}$: C 54.36, H 3.16, N 14.63. Found: C 54.42, H 3.50, N 14.89.

**Synthesis of $(Me_2NH_2)[Zn_2(L)(H_2O)]\cdot 4DMA$ (1)**

A mixture containing 0.04 mmol $H_5L$ and 0.04 mmol $Zn(NO_3)_2\cdot 6H_2O$ in DMA/$H_2O$(2.4mL/1.6mL) in a 20 mL scintillation vial was heated at 115 ºC for 2 d and then cooled to room temperature. The colorless crystals were collected, washed with DMA and dried in air (55.4% yield). Element analysis (%) calcd for $C_{44}H_{59}N_{11}O_{15}Zn_2$: C, 47.50; H, 5.34; N, 13.84. Found: C, 47.25; H, 5.29; N, 13.63. IR ($cm^{-1}$): 3134(vs), 2941(m), 1596(m), 1500(s), 1402(s), 1348(s), 1289(m), 1268(m), 1228(m), 1185(w), 1013(w), 808(w), 768(m).

**Procedure for encapsulating luminescent cations into 1.**

In each trial, 50 mg crystals of **1** were soaked in a 50 mL MeOH solution containing $[Ir(CF_3\text{-ppy-}F_2)_2(bpy)]PF_6$, $[Ru(bpy)_3](PF_6)_2$ or both at systematically varied concentrations. The mixture was stirred at ambient temperature for 48 h and subsequently filtered off to afford a solid and a filtrate. The amount of encapsulated cations were calculated from the difference between the amount in the starting solution and that in the filtrate; the concentration of the filtrate was measured by luminescent measurement. The solid was dried in air for further characterization.

**Luminescence sensing of solvents and some aromatic compounds.**

Each detection was repeated at least three times and consistent results were obtained. The solvents used in this study were N,N-dimethylacetamide (DMA), N, N-dimethylformamide (DMF), dimethyl sulfoxide

(DMSO), methanol (MeOH), tetrahydrofuran(THF), acetonitrile (MeCN), cyclohexane (CYH), acetone, $CH_2Cl_2$ (DCM) and ethylene glycol (EG). Th**e** aromatic compounds used in this study were benzene, fluorobenzene (F-benzene), chlorobenzene (Cl-benzene), bromobenzene (Br-benzene), tolune, *m*-xylene and *p*-xylene. Each time 10 mg finely-ground powder of **W2** was dispersed in 10 mL solvent, subjected to ultrasonication for 1 h and then aged for 3 h to form a stable emulsion for the luminescent studies.

**Luminescence sensing of NAC vapors.**

Each detection was repeated at least three times and consistent results were obtained. The NACs used in this study were nitrobenzene (NB), 1,2-dinitrobenzene (*o*-DNB), 1,3-dinitrobenzene (*m*-DNB), 1,4-dinitrobenzene (*p*-DNB), 4-nitrotoluene (4-NT), α-nironaphthalene (1-NP), 1-bromo-4-nitrobenzene (4-NBB), picric acid (TNP), dinoseb, o-nitrophenol (2-NPHEN), p-nitrophenol (4-NPHEN). Each time 0.5 mL MeOH and 3 mg finely-grounded powders of **W2** were mixed on a quartz plate to get a slurry which was left dry to afford a thin layer of **W2**. In parallel, a 5 mL open glass vial containing 100 mg solid NAC (1,2-DNB, 1,3-DNB, 1,4-DNB, 4-NT, 1-NP, 4-NBB, TNP, dinoseb, 2-NPHEN or 4-NPHEN) or 1 mL of NB was put into a sealed glass cuvette for a few days to ensure that the NAC's vapor pressure reached the equilibrium. Then the quartz plate was put into the sealed glass cuvette for a specific period of time and taken out for immediate luminescent measurements. The quenching efficiency (%) was estimated using the formula $[(I_0-I)/I_0]\times100\%$.

**X-ray Crystallographic Study**

The intensity data was collected at 153(2) K on a Mar CCD 165 mm detector by ω-scan techniques on the beamline 3W1A of BSRF (Beijing Synchrotron Radiation Facility) at the wavelength of 0.75000 Å. Data were corrected for absorption effects using the spherical harmonics technique. The structures were solved by direct methods using SHELXS-97 and were refined by full-matrix least squares methods using

SHELXL-97.[4] The hydrogen atoms were included in the structural model as fixed atoms "riding" on their respective carbon atoms using the idealized $sp^2$-hybridized geometry and C–H bond lengths of 0.95Å. No attempts were made to locate the hydrogen atoms on the coordinated $H_2O$. Since the $(Me_2NH_2)^+$ counter cations and the disordered DMA solvent molecules could not be unambiguously modelled, the PLATON/SQUEEZE[5] program was utilized to calculate the solvent disorder area and remove its contribution to the overall intensity data. The residual electron densities amounted to 216 e per formula, which roughly correspond to one $(Me_2NH_2)^+$ and 4 DMA molecules for **1**. A summary of the most important crystal and structure refinement data is given in Table S1. CCDC 1565928 contains the supplementary crystallographic data for this paper. The data can be obtained free of charge via www.ccdc.cam.ac.uk/conts/retrieving.html (or from the Cambridge Crystallographic Data Centre, 12 Union Road, Cambridge CB2 1EZ, UK; fax: (+44) 1223-336-033; or e-mail: deposit@ccdc.cam.ac.uk).

# Tables

**Table S1.** Crystallographic data for complex **1**.

| | **1** |
|---|---|
| Formula | $C_{44}H_{59}N_{11}O_{15}Zn_2$ |
| Formula weight | 1112.82 |
| Crystal system | Tetragonal |
| Space group | I4 |
| $a$ (Å) | 27.458(4) |
| $b$ (Å) | 27.458(4) |
| $c$ (Å) | 18.225(4) |
| $\alpha$ (°) | 90 |
| $\beta$ (°) | 90 |
| $\gamma$ (°) | 90 |
| $V$ (Å$^3$)/ $Z$ | 13741(5)/8 |
| $D_{calcd}$ (g /cm$^3$) | 0.694 |
| $\mu$(mm$^{-1}$) | 0.728 |
| $F$(000) | 2888 |
| $\theta$ range(°) | 2.47 -29.11 |
| Reflections collected / unique | 33549 / 9495 |
| $R$(int) | 0.0472 |
| GOF on $F^2$ | 1.106 |
| $R_1{}^a$, $I>2\sigma(I)$ (all) | 0.0323 (0.0330) |
| $wR_2{}^b$, $I>2\sigma(I)$ (all) | 0.0926 (0.0931) |
| Max/mean shift in final cycle | 0.001/0.000 |

$^aR=\Sigma(||F_o|-|F_c||)/\Sigma|F_o|$, $^bRw=\{\Sigma w[(F^2_o-F^2_c)]/\Sigma w[(F^2_o)^2]\}^{0.5}$, $w = [\sigma^2(F^2_o) + (aP)^2+bP]^{-1}$, where $P = (F^2_o +2 F^2_c)/3$. ]. **1**, $a$ = 0.0586, $b$ = 1.2152.

**Table S2. Bond lengths (Å) and angles (°) for 1.**

| | | | |
|---|---|---|---|
| Zn(1)-O(1) | 1.9332(15) | Zn(2)-O(10) | 2.0458(19) |
| Zn(1)-O(6)#1 | 1.9374(17) | Zn(2)-O(11) | 2.222(4) |
| Zn(1)-O(3)#2 | 1.9406(17) | O(3)-Zn(1)#3 | 1.9406(17) |
| Zn(1)-O(9) | 1.9920(19) | O(4)-Zn(2)#3 | 2.0160(16) |
| Zn(2)-O(7)#2 | 1.9532(17) | O(5)-Zn(2)#4 | 1.978(2) |
| Zn(2)-O(5)#1 | 1.978(2) | O(6)-Zn(1)#4 | 1.9374(17) |
| Zn(2)-O(4)#2 | 2.0160(16) | O(7)-Zn(2)#3 | 1.9533(17) |

| | | | |
|---|---|---|---|
| O(1)-Zn(1)-O(6)#1 | 107.71(9) | O(5)#1-Zn(2)-O(4)#2 | 121.92(8) |
| O(1)-Zn(1)-O(3)#2 | 107.19(7) | O(7)#2-Zn(2)-O(10) | 95.62(8) |
| O(6)#1-Zn(1)-O(3)#2 | 120.56(9) | O(5)#1-Zn(2)-O(10) | 101.99(9) |
| O(1)-Zn(1)-O(9) | 98.99(7) | O(4)#2-Zn(2)-O(10) | 93.02(9) |
| O(6)#1-Zn(1)-O(9) | 112.91(8) | O(7)#2-Zn(2)-O(11) | 86.57(12) |
| O(3)#2-Zn(1)-O(9) | 107.20(9) | O(5)#1-Zn(2)-O(11) | 80.04(12) |
| O(7)#2-Zn(2)-O(5)#1 | 129.52(8) | O(4)#2-Zn(2)-O(11) | 81.90(14) |
| O(7)#2-Zn(2)-O(4)#2 | 103.65(7) | O(10)-Zn(2)-O(11) | 174.84(14) |

**Symmetry transformations used to generate equivalent atoms:**

#1: y-1/2, -x+1/2, z+1/2; #2: -y+1, x, z; #3: -y+1/2, x+1/2, z-1/2; #4: y, -x+1, z.

## Figures

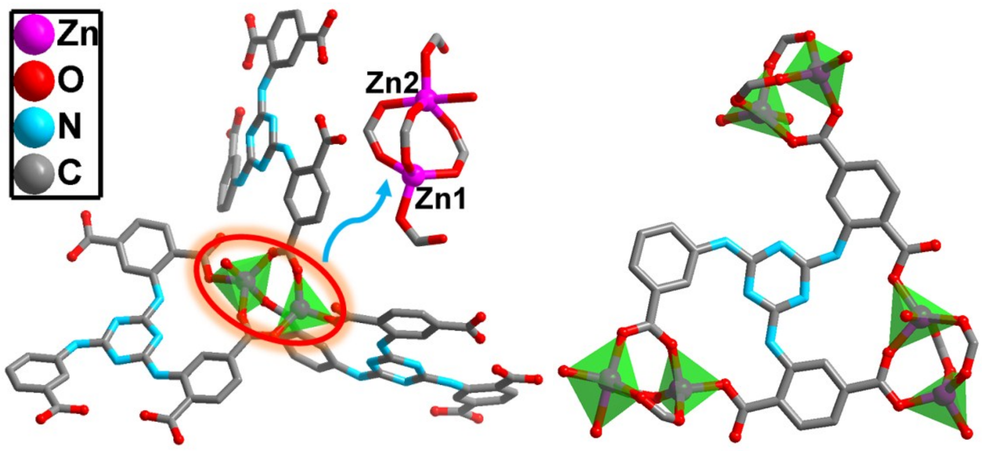


(a) (b)

**Figure S1.** The structure of compound **1**. (a) The coordination environment of the dinuclear quasi-paddlewheel $\{Zn_2(COO)_5\}$ molecular building block (MBB); (b) The bridging mode for the ligand to connect three MBBs.

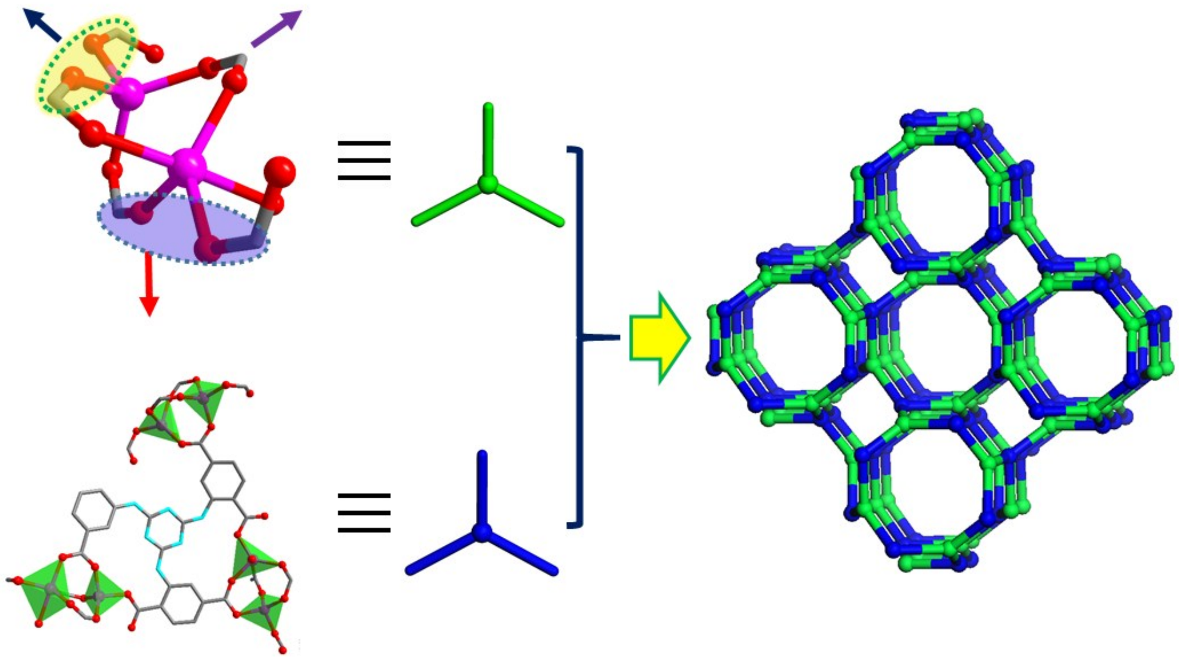

**Figure S2.** Schematic representation of the (3, 3)-connected *bcu-f* topological network of **1**.

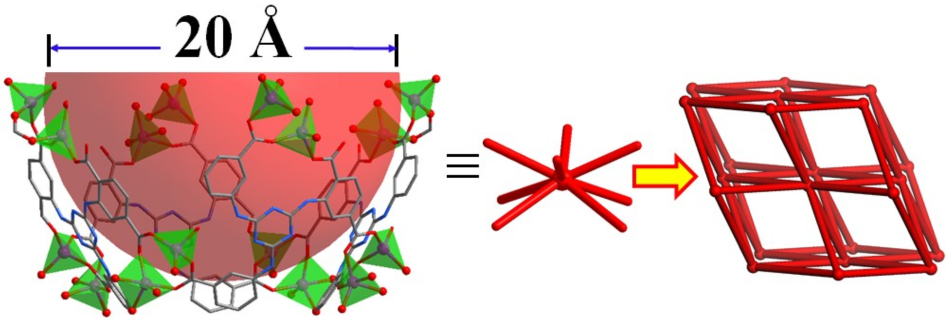


**Figure S3.** Schematic representation of the 8-connected *bcu* topological network of **1** when each $\{Zn_{16}L_4\}$ SBB is viewed as an eight-connected node .

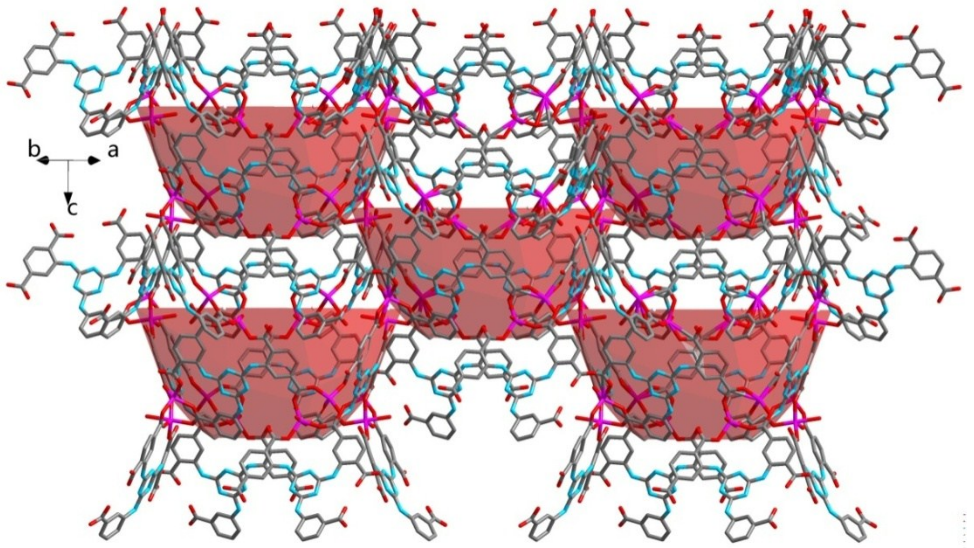


**Figure S4.** The packing of the framework viewed along the [1 1 0] direction.

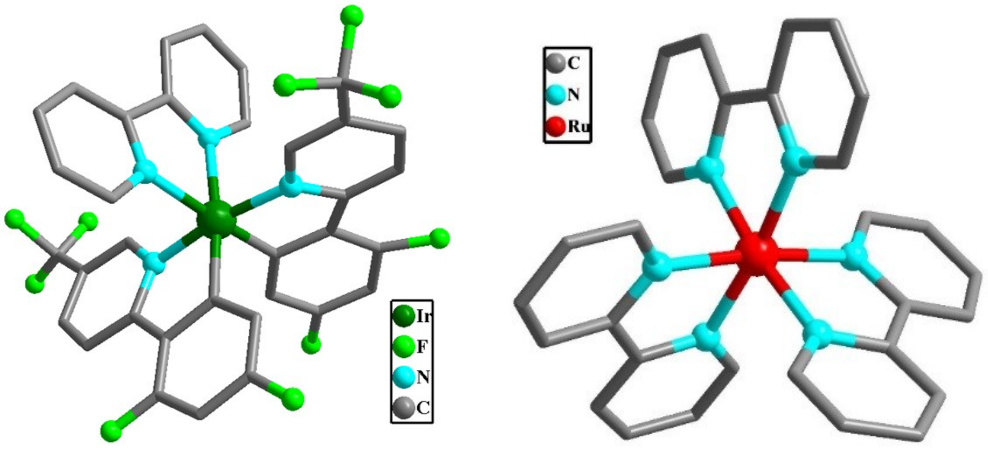


(a) (b)

**Figure S5.** (a) The structure of the $[Ir(CF_3\text{-ppy-}F_2)_2(bpy)]^+$ cation in $[Ir(CF_3\text{-ppy-}F_2)_2(bpy)]PF_6$; (b) The structure of the $[Ru(bpy)_3]^{2+}$ cation in $[Ru(bpy)_3](PF_6)_2$.

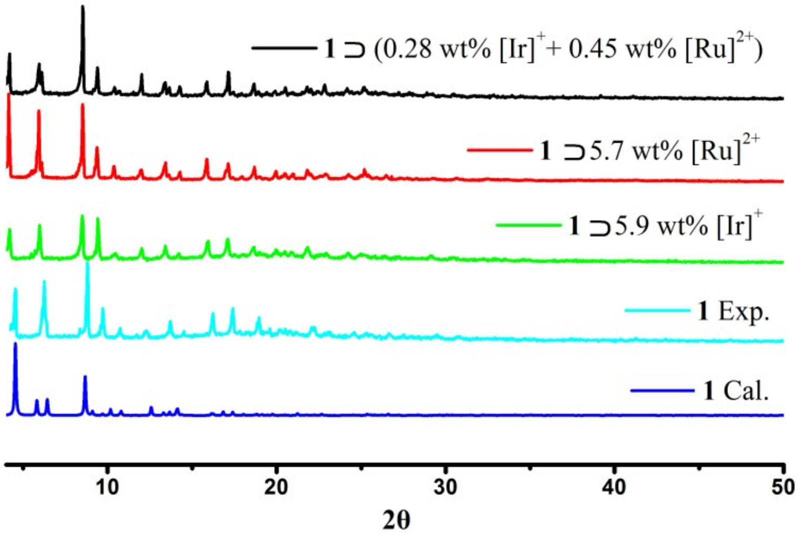


**Figure S6.** Experimental and simulated powder X-ray diffraction patterns of **1**, **G1**(**1**⊃ 5.9 wt% $[Ir]^{+}$), **R1**(**1**⊃ 5.7 wt% $[Ru]^{2+}$) and **W2** (**1**⊃ (0.28 wt% $[Ir]^{+}$+ 0.45 wt% $[Ru]^{+}$)). **Gx**, **Ry** and **Wz** represent the three series of composites, **1⊃$[Ir]^{+}$**, **1⊃$[Ru]^{2+}$** and **1⊃ ($[Ir]^{+}$+$[Ru]^{2+}$)** containing different amount of $[Ir]^{+}$ and /or $[Ru]^{2+}$ respectively.

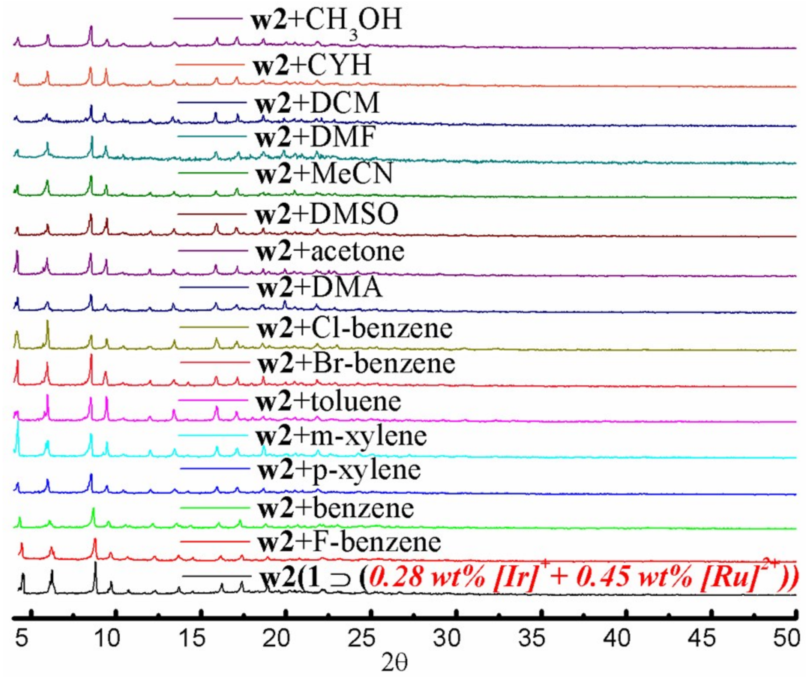


**Figure S7.** PXRD analyses indicate that **W2** retains its framework when being soaked in different VOCs at room temperature.

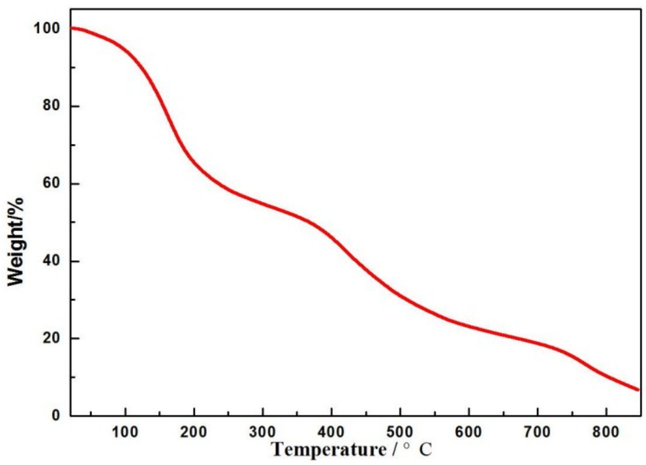


**Figure S8.** TGA curve of **1**.

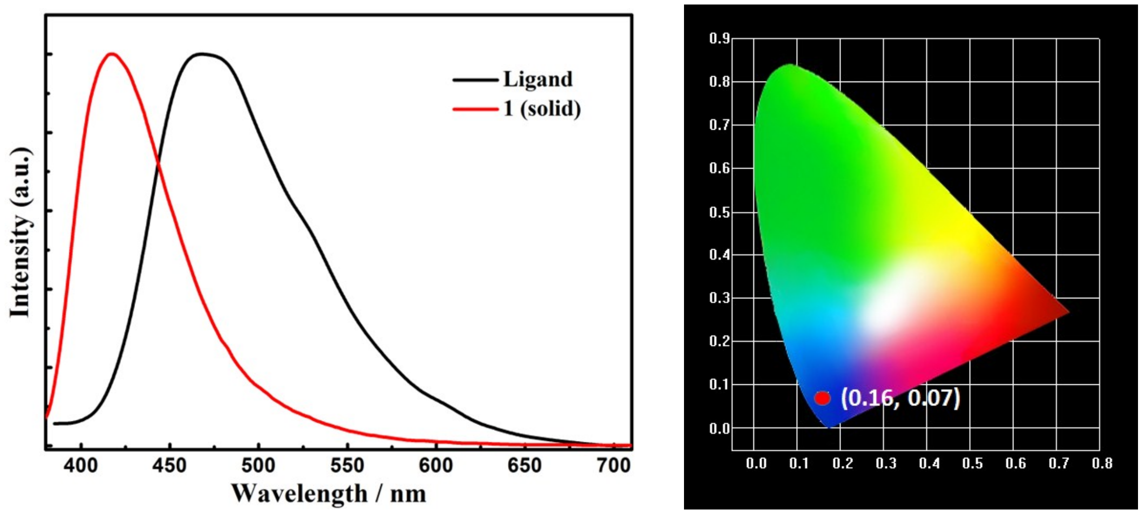


(a) (b)

**Figure S9** (a) Solid-state luminescent spectra of ligand $H_5L$ and compound **1** under $\lambda_{ex}$ = 365 nm at room temperature and (b) CIE chromaticity coordinates of **1**.

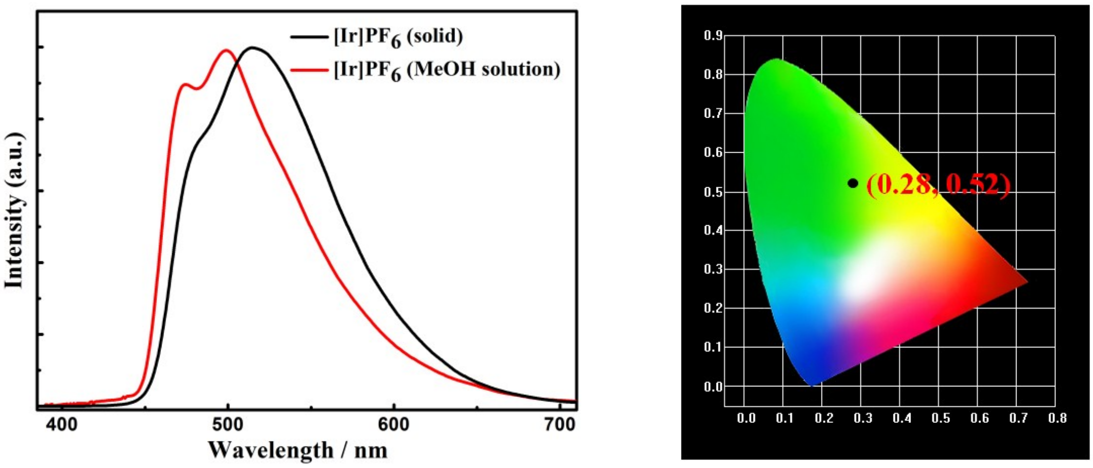


(a) (b)

**Figure S10.** (a) Luminescent spectra of $[Ir(CF_3\text{-ppy-}F_2)_2(bpy)]PF_6$ in the solid state and in MeOH solution at room temperature ($\lambda_{ex}$ = 365 nm) and (b) CIE chromaticity coordinates of solid-state $[Ir(CF_3\text{-ppy-}F_2)_2(bpy)]PF_6$.

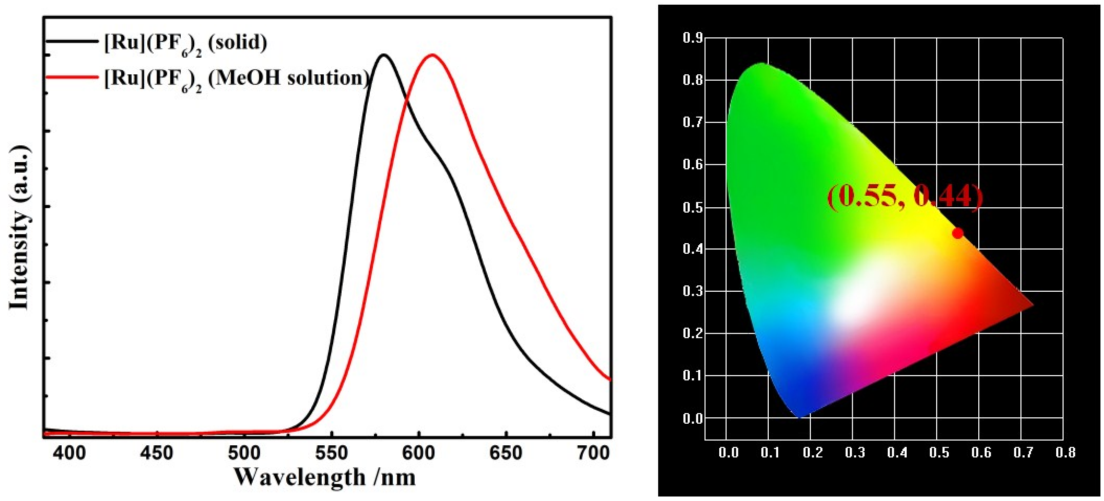


(a) (b)

**Figure S11.** (a) Luminescent spectra of $[Ru(bpy)_3](PF_6)_2$ in the solid state and in MeOH solution at room temperature ( $\lambda_{ex}$ = 365 nm) and (b) CIE chromaticity coordinates of solid-state $[Ru](PF_6)_2$.

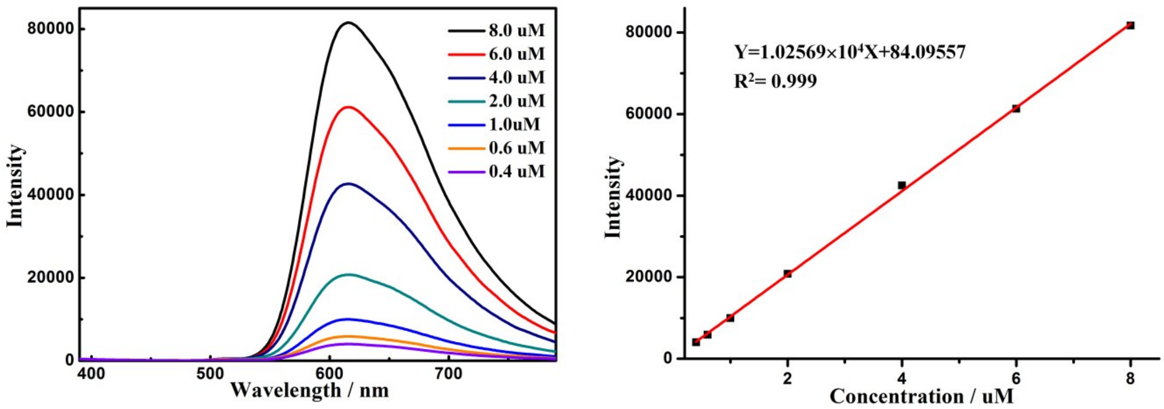


(a) (b)

**Figure S12**. (a) Luminescent spectra of MeOH solutions of $[Ru(bpy)_3](PF_6)_2$ at different concentrations at room temperature ($\lambda_{ex}$ = 365 nm). (b) The linear correlation between the luminescent intensity (monitored at 614 nm) and the $[Ru(bpy)_3](PF_6)_2$ concentration.

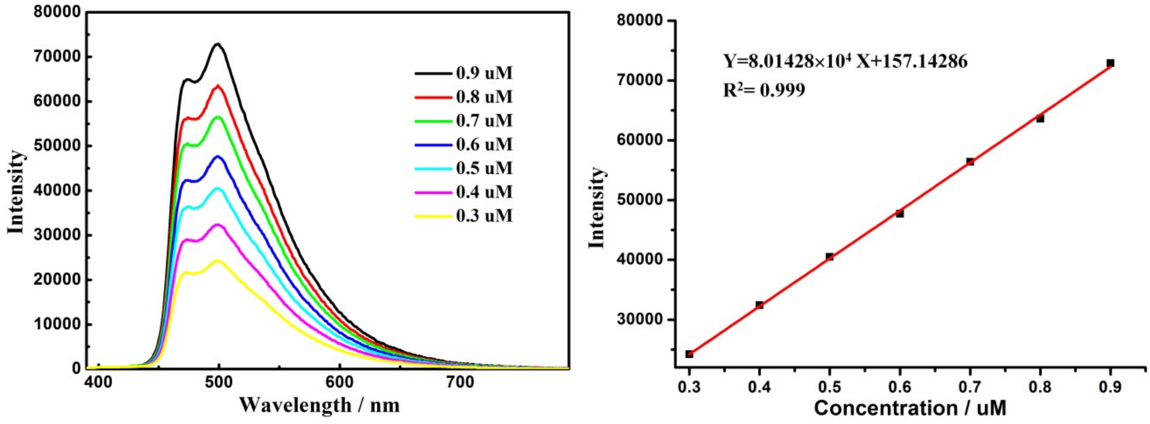


(a) (b)

**Figure S13**. (a) Luminescent spectra of MeOH solutions of $[Ir(CF_3\text{-}ppy\text{-}F_2)_2(bpy)]PF_6$ at different concentrations at room temperature ($\lambda_{ex}$ = 365 nm). (b) The linear correlation between the luminescent intensity (monitored at 498 nm) and the $[Ir(CF_3\text{-}ppy\text{-}F_2)_2(bpy)]PF_6$ concentration.

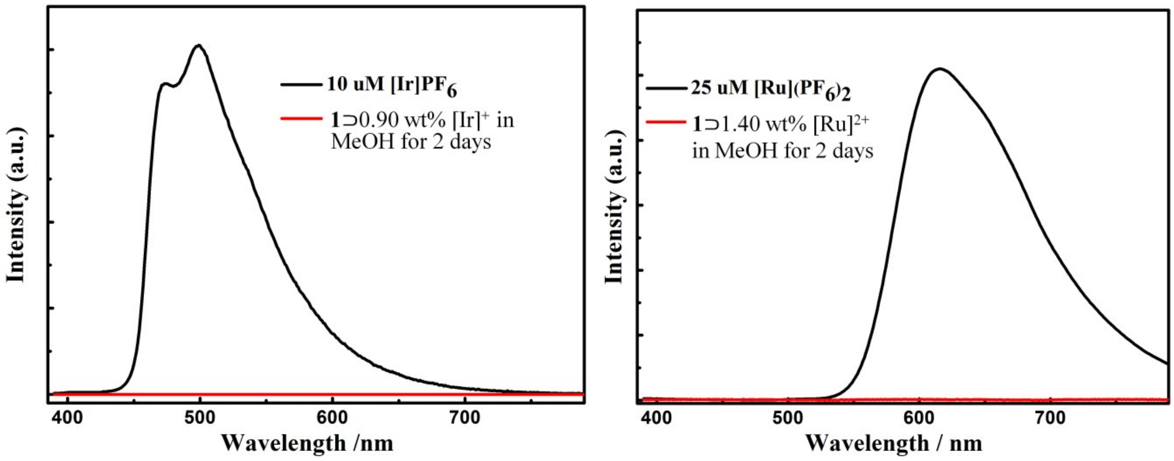


(a) (b)

**Figure S14.** (a) Emission spectra of 10 μM $[Ir(CF_3\text{-}ppy\text{-}F_2)_2(bpy)]PF_6$ in MeOH and the filtrate after immersing the as-synthesized **1**⊃0.9 wt% $[Ir(CF_3\text{-}ppy\text{-}F_2)_2(bpy)]^+$ into MeOH for 2 days at room temperature ( $\lambda_{ex}$ = 365 nm). (b) Emission spectra of 25 μM $[Ru(bpy)_3](PF_6)_2$ in MeOH and the filtrate after immersing the as-synthesized **1**⊃1.4 wt% $[Ru]^{2+}$ into MeOH for 2 days at room temperature ( $\lambda_{ex}$ = 365 nm).

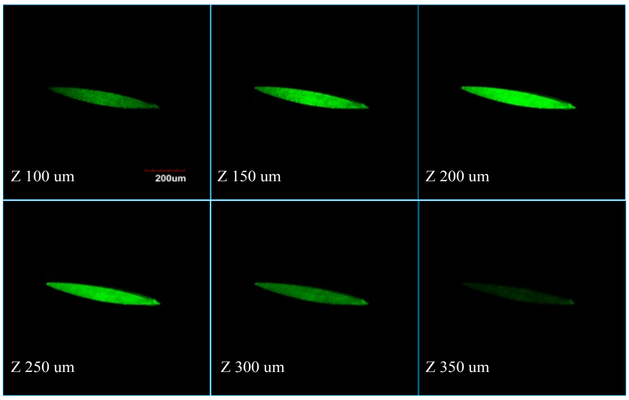


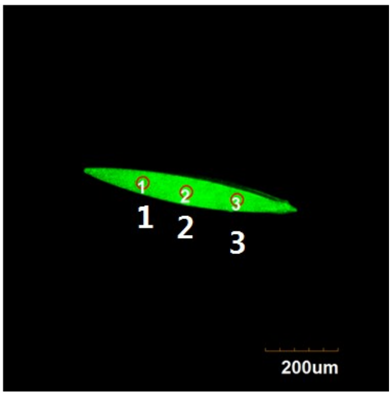


(a) (b)

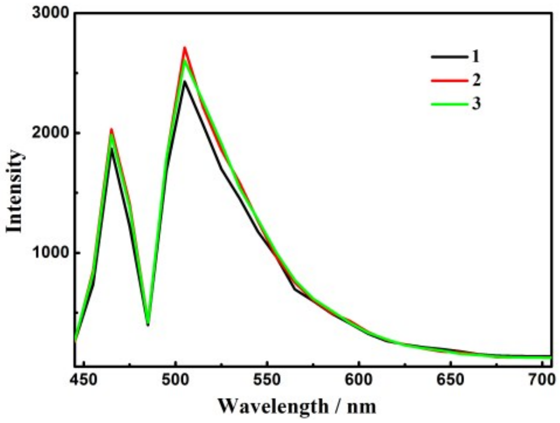


(c)

**Figure S15.** (a–b) Confocal scanning images of a sample obtained by soaking single crystal of **1** into a MeOH solution of [Ir($CF_3$-ppy-$F_2$)$_2$(bpy)]$PF_6$ in the depth-scan mode collected at the wavelength of 440-710 nm (a) and under the lambda (wavelength) scan mode (λ=440-710 nm) (b). (c) Luminescent spectra of three selected regions in (b).

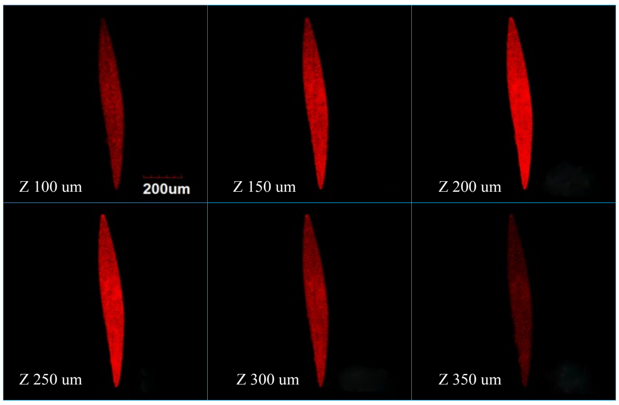


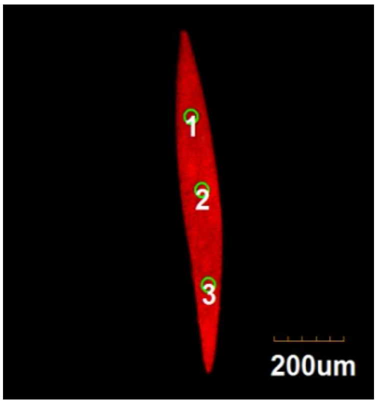


(a) (b)

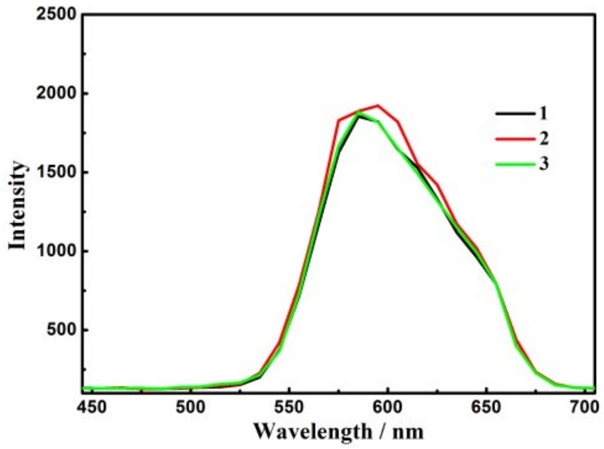


(c)

**Figure S16.** (a–b) Confocal scanning images of a sample obtained by soaking single crystal of **1** into a MeOH solution of $[Ru(bpy)_3](PF_6)_2$ in the depth-scan mode collected at the wavelength of 440-710 nm (a) and under the lambda (wavelength) scan mode (λ=440-710 nm) (b). (c) Luminescent spectra of three selected regions in (b).

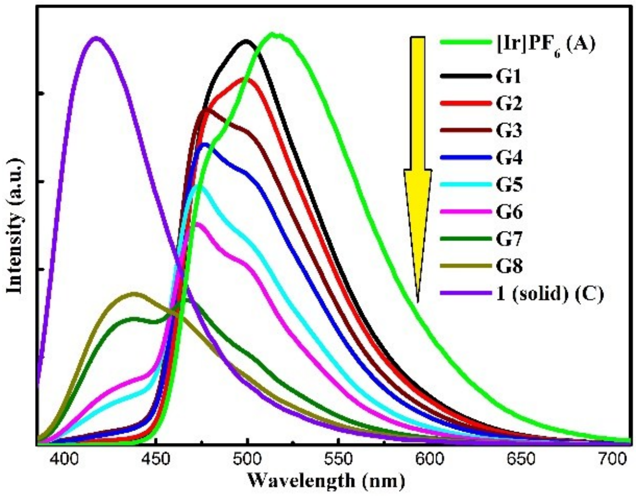


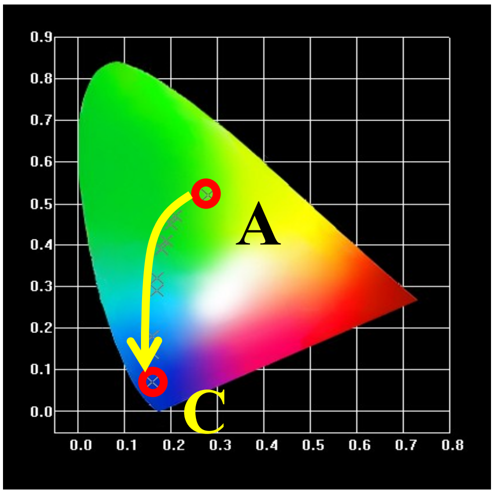


(a) (b)

**Figure S17**. Luminescent spectra (a) and CIE chromaticity coordinates (b) of **1**, $[Ir(CF_3\text{-ppy-}F_2)_2(bpy)]PF_6$ and **1**⊃$[Ir]^+$ with different amounts of $[Ir]^+$ ($\lambda_{ex}$ = 365 nm) (**G1**: **1**⊃5.90 wt% $[Ir]^+$; **G2**: **1**⊃4.40 wt% $[Ir]^+$;

**G3**: **1**$\supset$2.10 wt% $[Ir]^+$; **G4**: **1**$\supset$1.80 wt% $[Ir]^+$; **G5**: **1**$\supset$0.70 wt% $[Ir]^+$; **G6**: **1**$\supset$ 0.35 wt% $[Ir]^+$; **G7**: **1**$\supset$0.070 wt% $[Ir]^+$; **G8**: **1**$\supset$0.018 wt% $[Ir]^+$) ($\lambda_{ex}$ = 365 nm).

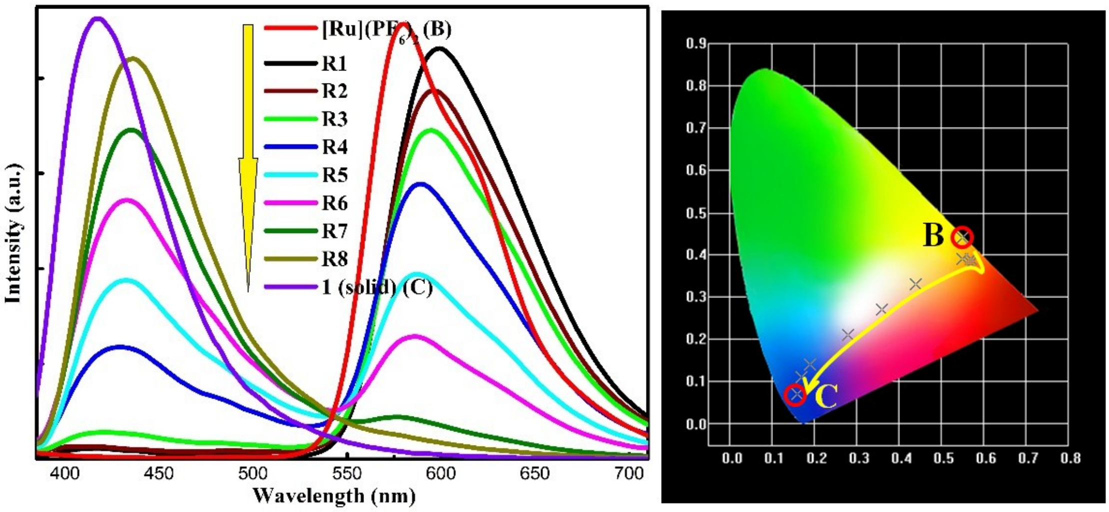


(a) (b)

**Figure S18.** Luminescent spectra (a) and CIE chromaticity coordinates (b) of **1**, $[Ru(bpy)_3](PF_6)_2$ and **1**$\supset[Ru]^{2+}$ with different amounts of $[Ru]^{2+}$ ($\lambda_{ex}$ = 365 nm) (**R1**: **1**$\supset$5.70 wt% $[Ru]^{2+}$; **R2**: **1**$\supset$4.60 wt% $[Ru]^{2+}$; **R3**: **1**$\supset$2.80 wt% $[Ru]^{2+}$; **R4**: **1**$\supset$1.10 wt% $[Ru]^{2+}$; **R5**: **1**$\supset$0.45 wt% $[Ru]^{2+}$; **R6**: **1**$\supset$ 0.23 wt% $[Ru]^{2+}$; **R7**: **1**$\supset$0.05 wt% $[Ru]^{2+}$; **R8**: **1**$\supset$0.01 wt% $[Ru]^{2+}$) ($\lambda_{ex}$ = 365 nm).

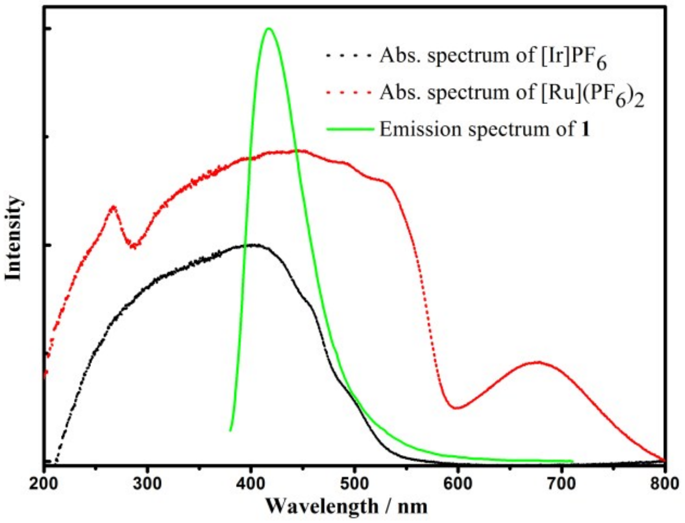


**Figure S19.** Spectral overlaps between the solid state absorption spectra of $[Ir(CF_3\text{-}ppy\text{-}F_2)_2(bpy)]PF_6$, $[Ru(bpy)_3](PF_6)_2$ and the emission spectrum of **1**.

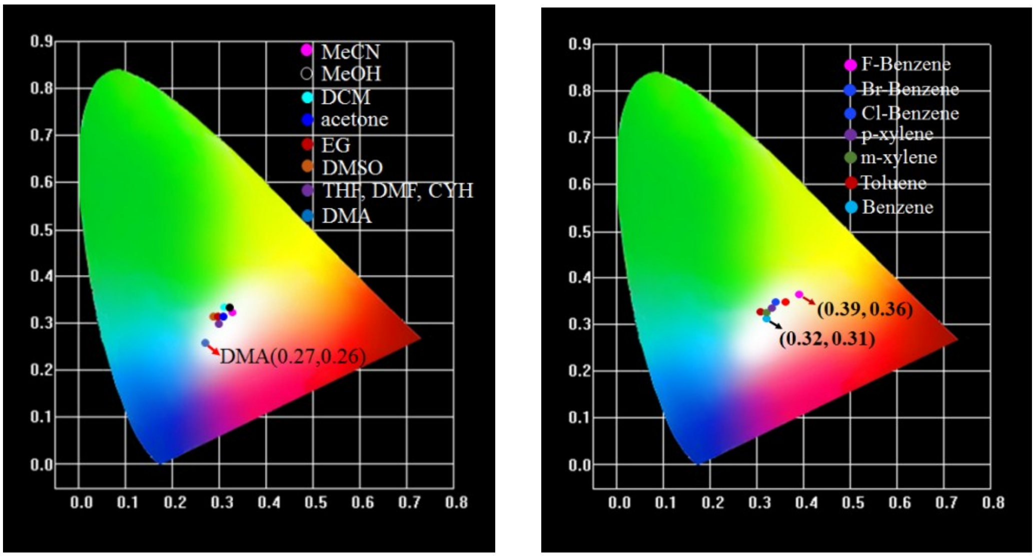


(a) (b)

**Figure S20.** The CIE coordinates of **W2** after the encapsulation of different solvent molecules (a) and aromatic molecules (b) ($\lambda_{ex}$ = 365 nm).

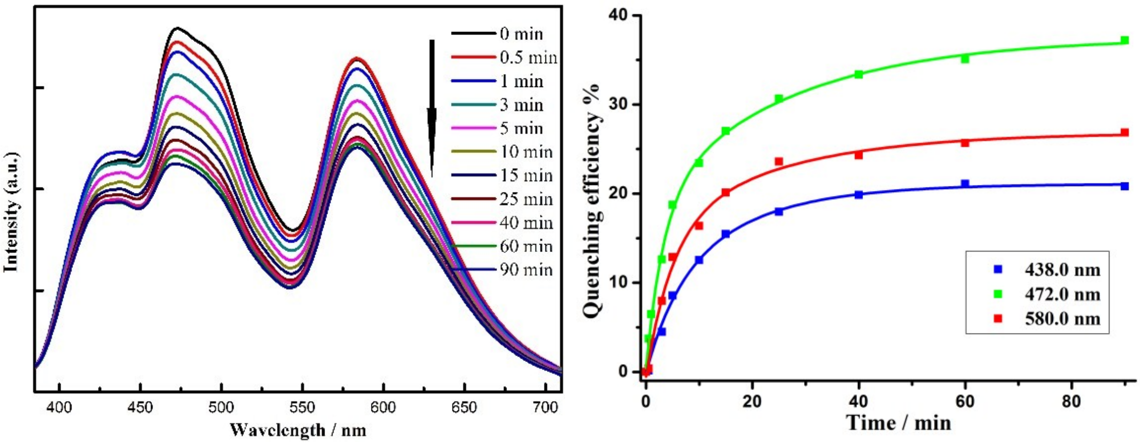


(a) (b)

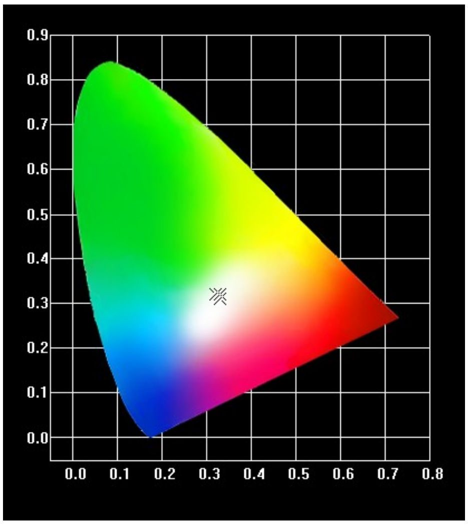

(c)

**Figure S21.** (a) and (b) are the time-dependent luminescent spectra and quenching efficiency of **W2** composite upon exposure to NB vapor at 25°C respectively. (c) CIE coordinate for each spectrum in (a).

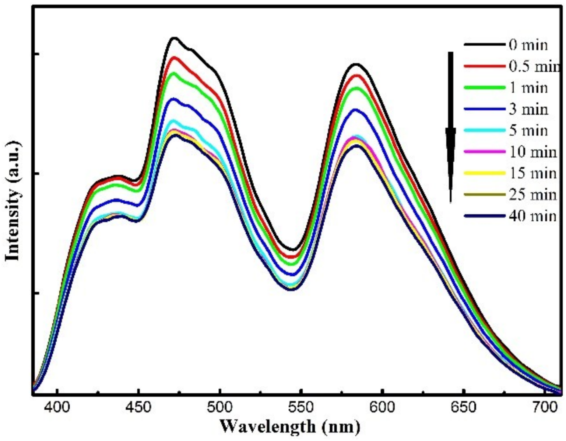


(a)

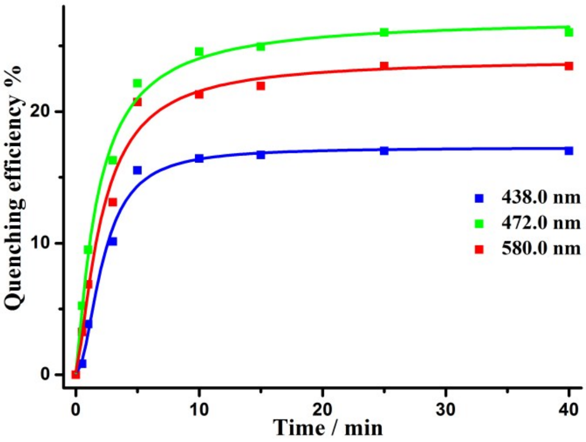


(b)

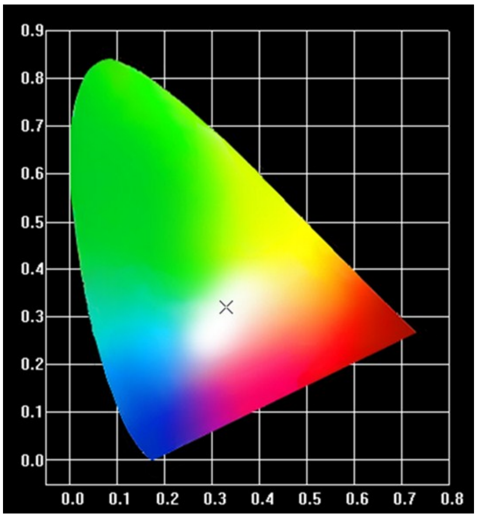


(c)

**Figure S22.** (a) and (b) are the time-dependent luminescent spectra and quenching efficiency of **W2** composite upon exposure to NB vapor at 40°C respectively. (c) CIE coordinate for each spectrum in (a).

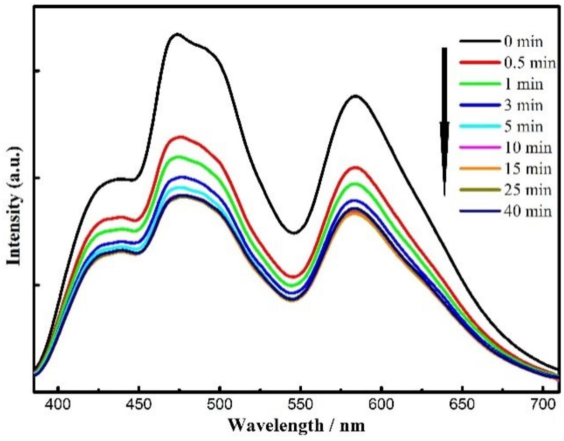


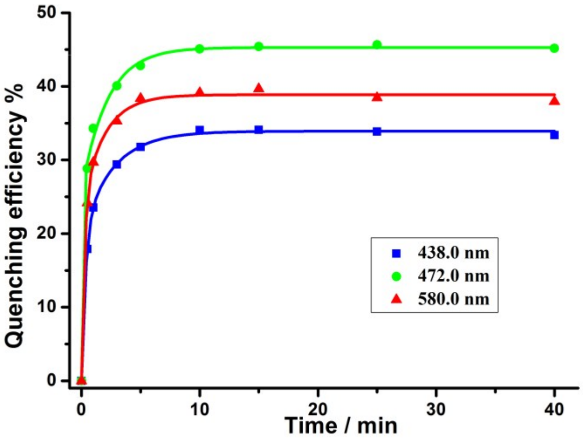


(a) (b)

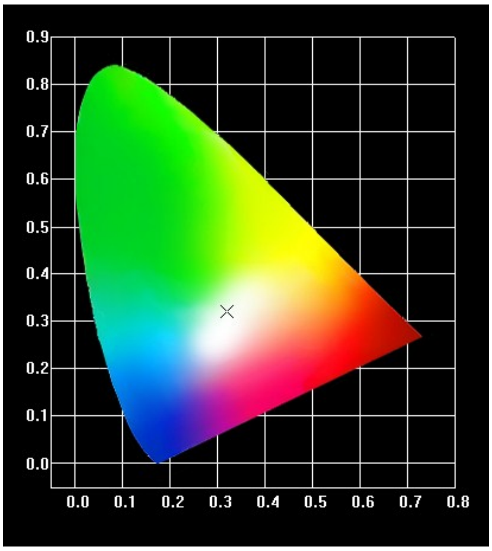


(c)

**Figure S23.** (a) and (b) are the time-dependent luminescent spectra and quenching efficiency of **W2** composite upon exposure to NB vapor at 60°C respectively. (c) CIE coordinate for each spectrum in (a).

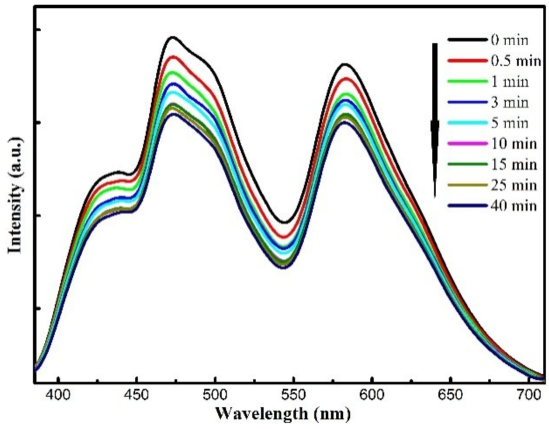


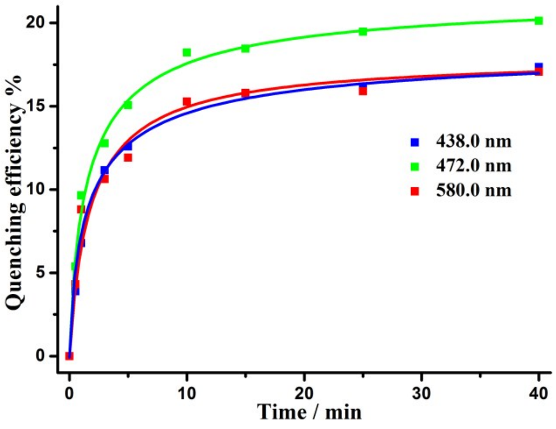


(a) (b)

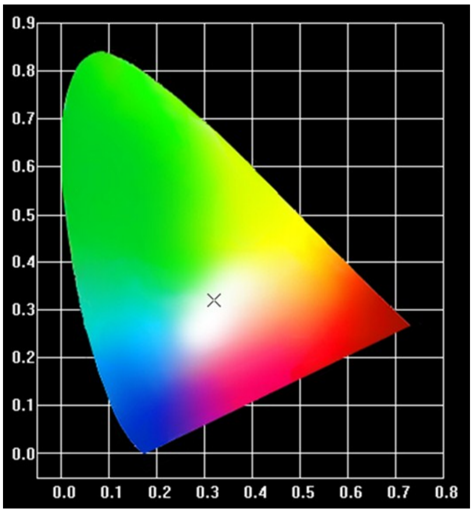


(c)

**Figure S24.** (a) and (b) are the time-dependent luminescent spectra and quenching efficiency of **W2** composite upon exposure to *o*-DNB vapor at 40°C respectively. (c) CIE coordinate for each spectrum in (a).

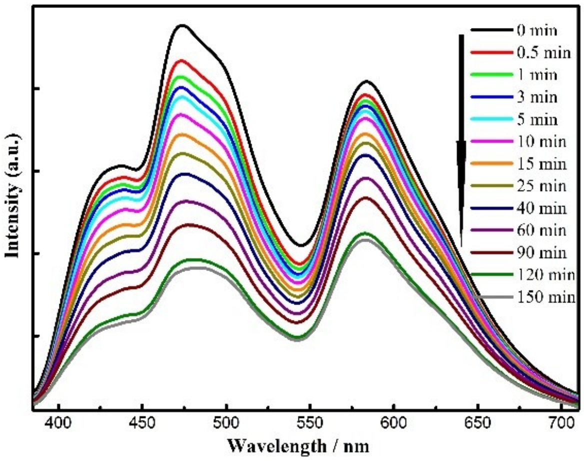


(a)

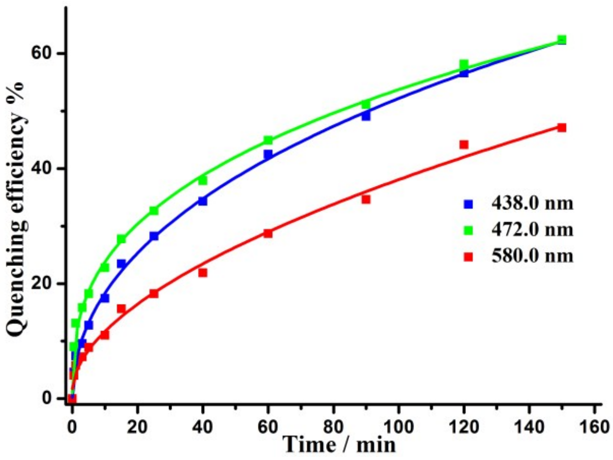


(b)

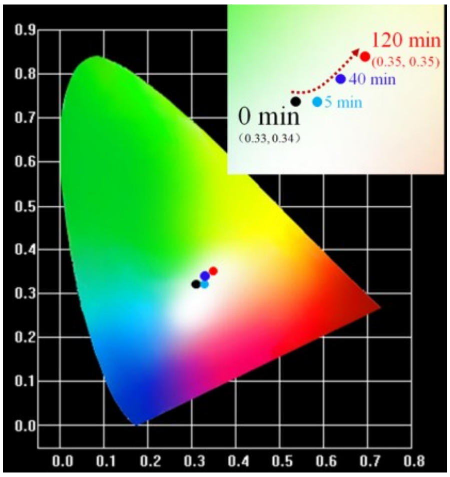


(c)

**Figure S25.** (a) and (b) are the time-dependent luminescent spectra and quenching efficiency of **W2** composite upon exposure to *o*-DNB vapor at 60°C respectively. (c) CIE coordinate for each spectrum in (a).

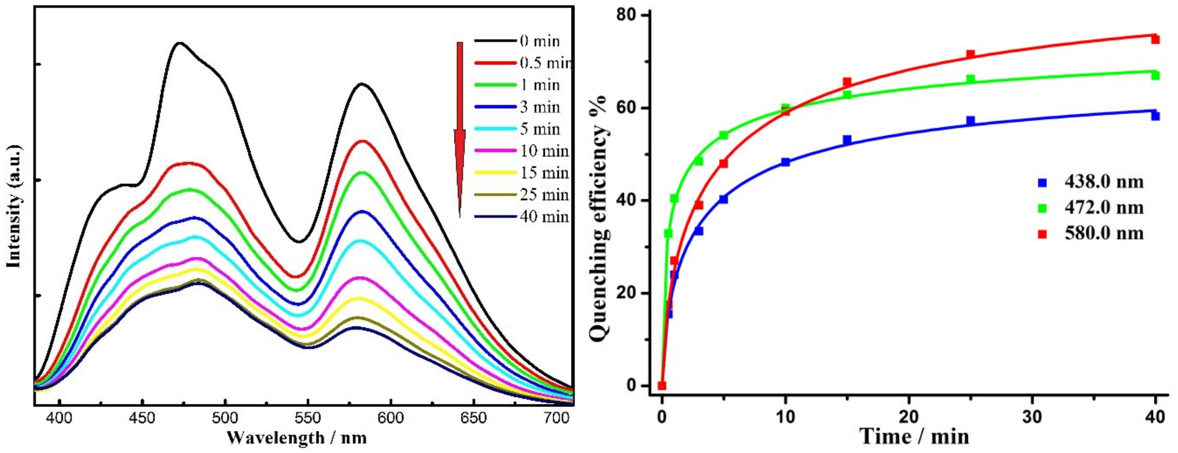


(a) (b)

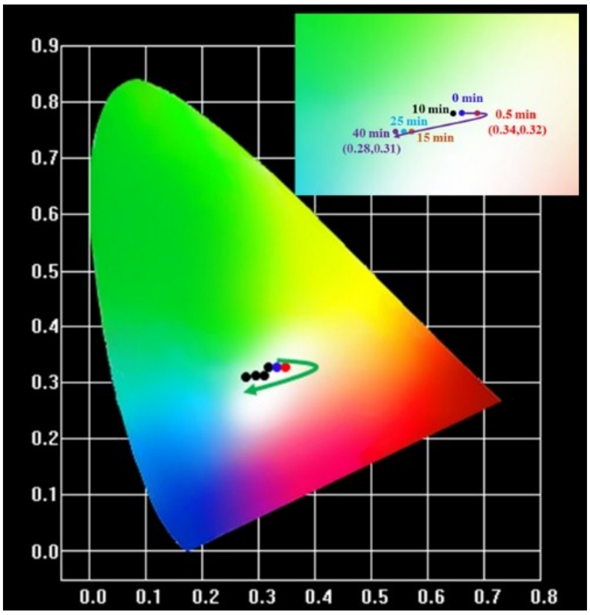


(c)

**Figure S26.** (a) and (b) are the time-dependent luminescent spectra (a) and quenching efficiency (b) of **W2** composite upon exposure to *m*-DNB vapor at 40°C respectively. (c) CIE coordinate for each spectrum in (a).

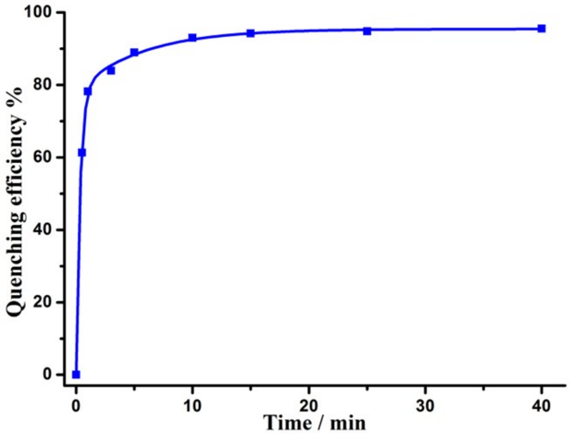


(a)

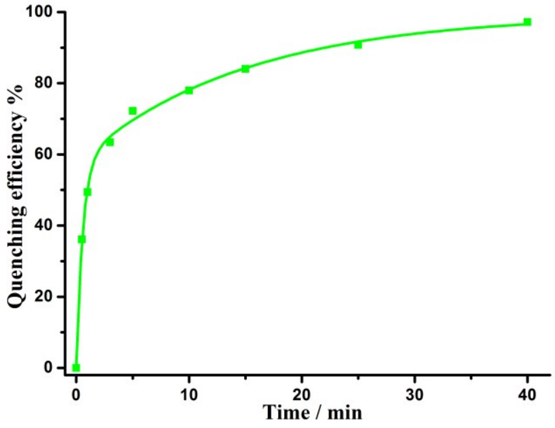


(b)

**Figure S27.** (a) The quenching efficiency of **1** upon exposure to *m*-DNB vapor at 60°C. (b) The quenching efficiency of $[Ir(CF_3\text{-ppy-}F_2)_2(bpy)]PF_6$ upon exposure to *m*-DNB vapor at 60°C.

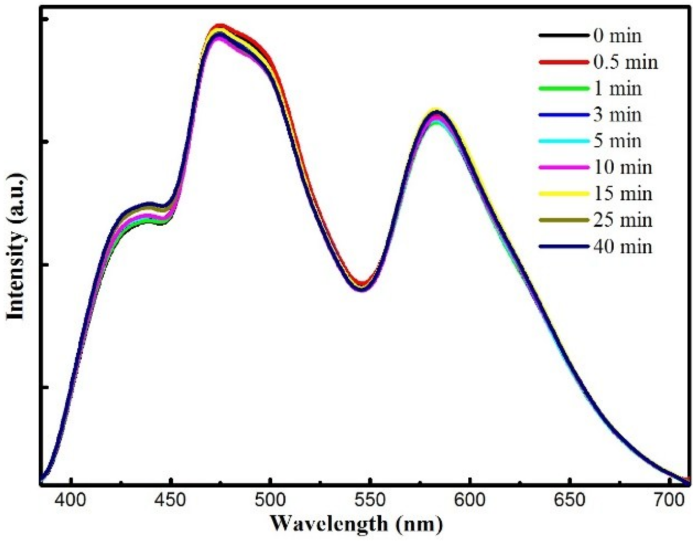


**Figure S28.** Time-dependent luminescent spectra of **W2** composite upon exposure to *p*-DNB vapor at 25°C.

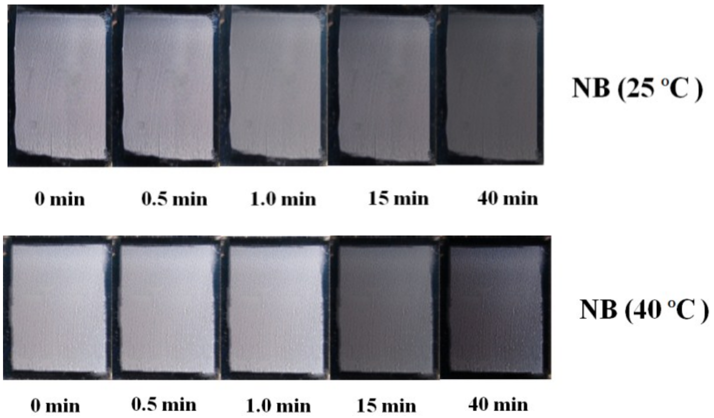

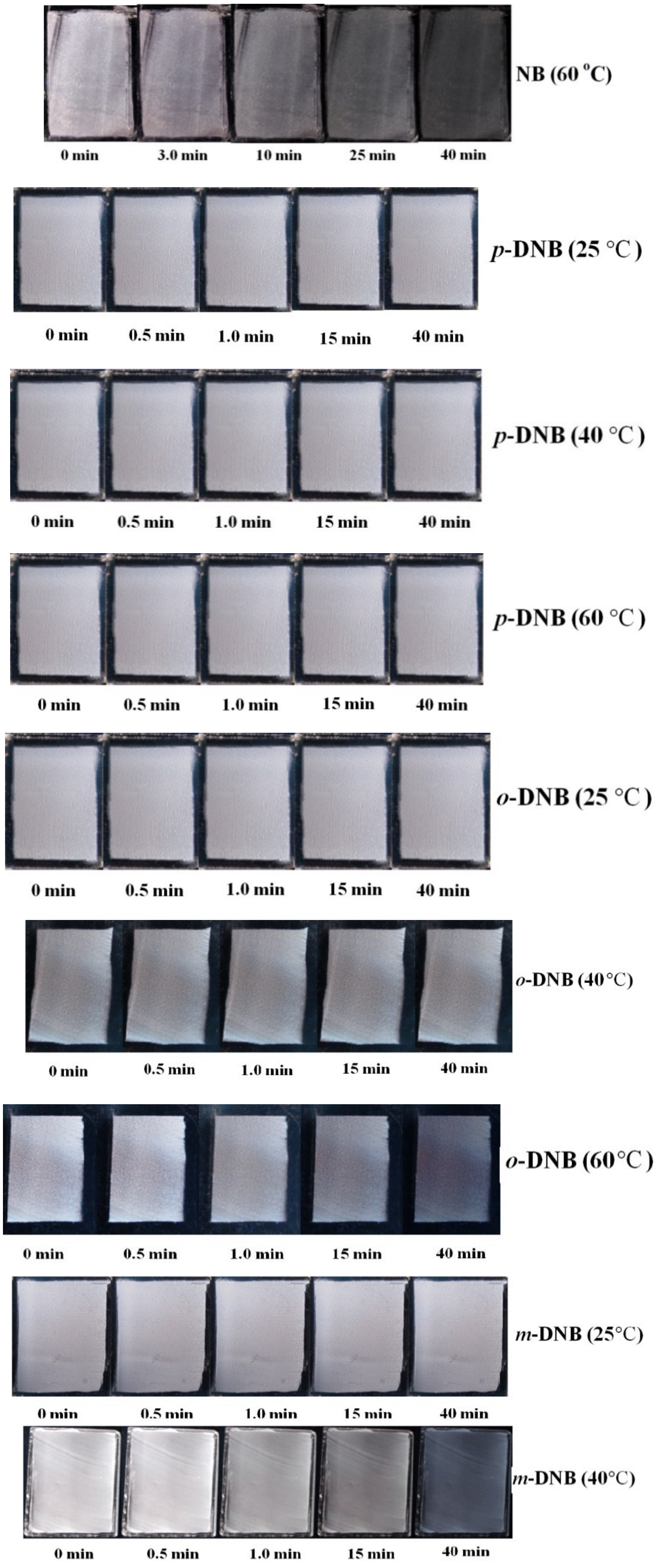
NB (60 °C)
0 min 3.0 min 10 min 25 min 40 min
p-DNB (25 °C)
0 min 0.5 min 1.0 min 15 min 40 min
p-DNB (40 °C)
0 min 0.5 min 1.0 min 15 min 40 min
p-DNB (60 °C)
0 min 0.5 min 1.0 min 15 min 40 min
o-DNB (25 °C)
0 min 0.5 min 1.0 min 15 min 40 min
o-DNB (40°C)
0 min 0.5 min 1.0 min 15 min 40 min
o-DNB (60°C)
0 min 0.5 min 1.0 min 15 min 40 min
m-DNB (25°C)
0 min 0.5 min 1.0 min 15 min 40 min
m-DNB (40°C)
0 min 0.5 min 1.0 min 15 min 40 min

**Figure S29**. Photographic images of **W2** upon exposure to different NAC vapors at different temperature and time under UV light irradiation (365 nm).

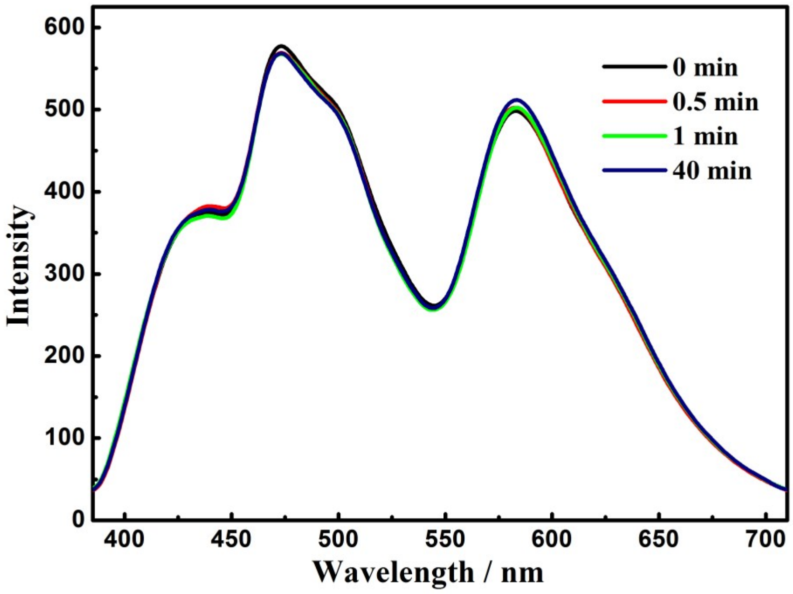


**Figure S30**. Time-dependent luminescent spectra of **W2** composite upon exposure to F-Benzene vapor at 60°C.

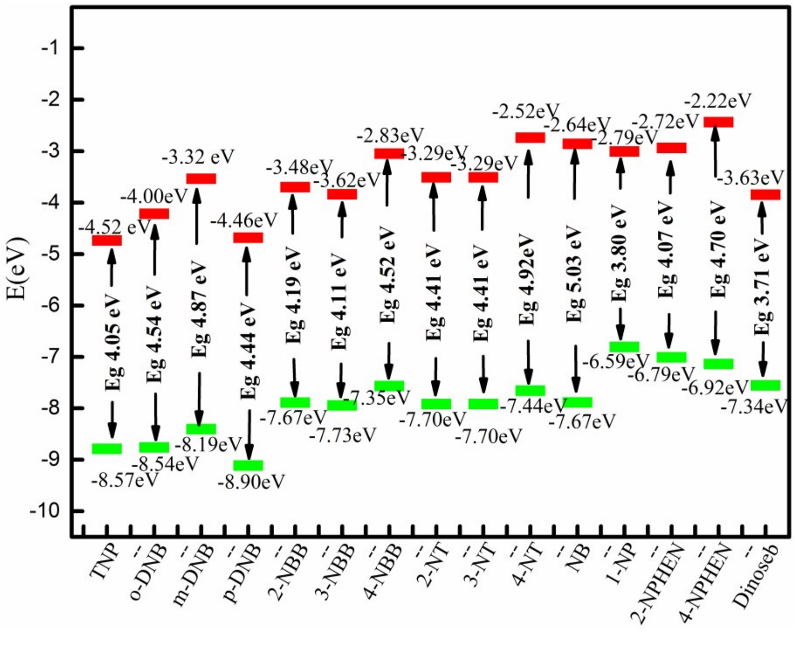


**Figure S31.** The HOMO and LUMO energy levels for all the NACs calculated with density functional theory (DFT) at the B3LYP/6-31G* level.

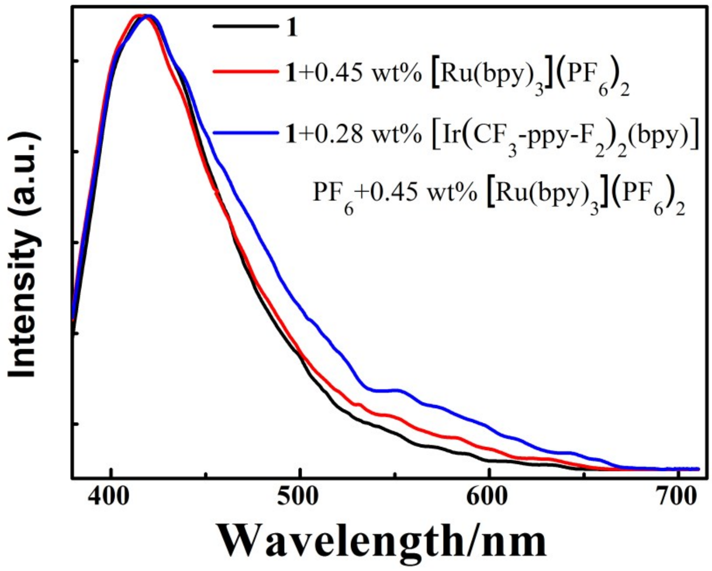


**Figure S32.** A comparsion of luminescent spectra of **1**, uniformly ground samples of **1**+0.45wt.%[Ru(bpy)$_3$](PF$_6$)$_2$ and **1**+0.45wt.%[Ru(bpy)$_3$](PF$_6$)$_2$+0.28wt.%[Ir(CF$_3$-ppy-F$_2$)$_2$ (bpy)]PF$_6$ in the solid state at room temperature ( $\lambda_{ex}$ = 365 nm).

## References

[1] D. Hanss, J.C. Freys, G. Bernardinelli, O.S. Wenger, *Eur. J. Inorg. Chem*., 2009, 4850–4859.

[2] J. Bouzaid, M. Schultz, Z. Lao, J. Bartley, T. Bostrom, J. McMurtrie, *Cryst. Growth Des*., 2012, **12**, 3906−3916.

[3] W. Xie, W.-W. He, D.-Y. Du, S.-L. Li, J.-S. Qin, Z.-M. Su, C.-Y. Sun, Y.-Q. Lan, *Chem. Commun*., 2016, **52**, 3288-3291.

[4] G. M. Sheldrick, SHELXL-97, Program for X-ray Crystal Structure Refinement, University of Göttingen: Göttingen, (Germany), 1997.

[5] A. L. Spek, PLATON, Multipurpose Crystallographic Tool, Utrecht University, Utrecht, The Netherlands, 2008.
